# Drivers of Stopover Distribution Across Two Autumn Migration Routes of a Long‐Distance Afro‐Palearctic Migrant, the Common Cuckoo ( *Cuculus canorus* )

**DOI:** 10.1002/ece3.74083

**Published:** 2026-08-04

**Authors:** Alex J. Gale, Máire Kirkland, Philip W. Atkinson, Robert Puschendorf, Chris M. Hewson

**Affiliations:** ^1^ School of Biological and Marine Sciences University of Plymouth Plymouth UK; ^2^ British Trust for Ornithology, The Nunnery Norfolk UK

## Abstract

Across the globe, many long‐distance migratory birds have undergone population declines. Understanding the environmental factors which underly migratory stopover locations can inform conservation strategies for such species. Here, we identify environmental predictors of autumn stopover sites used by UK‐breeding male cuckoos ( *Cuculus canorus* ) migrating to central African wintering grounds via two alternative flyways. Between 2011 and 2021, 88 male cuckoos from UK breeding grounds were fitted with < 5 g satellite tags connected to the Argos satellite‐based tracking system. Filtered stopovers were used to build presence‐pseudoabsence distribution models for each flyway, using Boosted Regression Tree and Random Forest algorithms with thirteen climatic, habitat, topographic and anthropogenic variables. Performance was evaluated using 10‐fold cross‐validation, and weighted ensembles were generated. To assess drought sensitivity, we produced predictions for high‐ and low‐drought years based on Standardised Precipitation–Evapotranspiration Indices (SPEI). Models performed well (AUC = 0.79–0.88; TSS = 0.50–0.66) and revealed route‐specific environmental predictors. Along the western route, stopover probability was highest in cooler, semi‐humid, high‐elevation areas with low contemporaneous drought; Iberian mountain ranges emerged as key sites. Along the eastern route, stopover probability was greatest in heterogeneous habitat, with lowlands and uplands favoured over intermediate elevations; the Po watershed appears particularly important. Hunting risk (included to assess anthropogenic threat) was also high at key areas. The spatial extent of stopover regions—and the probability of them being utilised—declined during years of high short‐term drought along the western route, and high long‐term drought along the eastern route. Whilst Iberian montane stopover regions are sensitive to increasing drought and aridity under climate change, their topographic heterogeneity may provide climatic refuge while limiting intensive land uses. Preference for heterogeneous habitat within the Po watershed highlights the need to retain semi‐natural habitat within agricultural landscapes. We recommend route‐specific conservation measures and higher resolution models using newly available GPS tags to better understand fine‐scale site selection within the regions highlighted here.

## Graphical abstract

We model autumn stopover distribution of UK common cuckoos across two migration routes, revealing distinct environmental drivers and key geographic hotspots. Iberian mountain systems and the Po watershed emerge as critical stopover regions, with climate, elevation, habitat heterogeneity, and hunting risk shaping route‐specific patterns. Increasing drought reduces stopover availability, underscoring growing climate vulnerability and the need for targeted conservation.

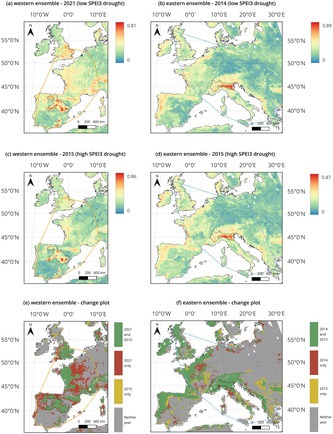

## 1 Introduction

Over the past 50 years, approximately half of the world's bird species have undergone population declines (Lees et al. 2022). This trend has been documented in a wide range of taxonomic groups with diverse life‐history traits; however, evidence from across the globe suggests that declines have been more pronounced in long‐distance migrants (Robbins et al. 1989; Ockendon et al. 2012; Vickery et al. 2014; Bayly et al. 2018; Rosenberg et al. 2019; Lees et al. 2022). Due to their complex annual cycles, long migration routes, and reliance on multiple sites at different times, migratory birds face ‘multiple jeopardies’ and are thus likely to be more susceptible to environmental change than resident species (Newton 2004a; Vickery et al. 2014). In Europe, where Afro‐Palearctic migrants breed before returning to wintering grounds in Africa, declines have been severe (Sanderson et al. 2006; Burns et al. 2021). Population trends for many Afro‐Palearctic migrants have been particularly well documented in the UK, where long‐term monitoring has captured the magnitude of declines at a species‐level (Stanbury et al. 2021). One such species, the Common Cuckoo ( *Cuculus canorus* , hereafter ‘cuckoo’) is amongst the most threatened UK migrants, having declined by 77% in England since 1965 (Heywood et al. 2023; BTO 2024). There is, however, regional variation in population trends, with the greatest declines in agricultural lowlands of south‐east England, less severe declines in upland areas such as Dartmoor, and even recent increases in the Scottish Highlands (Hewson et al. 2016; Denerley et al. 2019; Heywood et al. 2023).

A number of factors underlying cuckoo declines have been suggested. As brood parasites, cuckoos are reliant on hosts to rear their young and complete their reproductive cycle (Wyllie 1981). Cuckoo abundance and breeding success is, therefore, at least partially dependent on the availability of host nests. Nonetheless, evidence suggests that changes in host availability (either through population declines or phenological shifts) have not been principal drivers of historic population declines (Douglas et al. 2010). Cuckoos may face a variety of synergistic threats across all stages of their annual cycle (Buchan et al. 2023; Davies et al. 2023); amongst these, habitat loss and reduced abundance of invertebrate prey at UK breeding grounds are primary concerns (Newton 2004b; Denerley 2014; Bowler et al. 2019; Pearce‐Higgins and Morris 2023; Rigal et al. 2023). Indeed, dramatic declines in Lepidoptera larvae (Conrad et al. 2006; Fox et al. 2019), as well as Orthopteran and Dipteran prey species (Mills et al. 2020), have been concentrated in regions where cuckoo declines have been greatest; most notably, intensive agricultural lowlands of southern England (Denerley et al. 2019; Fox et al. 2021). Whilst poor conditions on breeding grounds can limit energy intake (Vickery et al. 2014; Fayet et al. 2021; Goodship and Furness 2022), mortality is often most acute during migratory periods (Newton 2025), where the combined effects of low fuel reserves, physiological stress and unfavourable stopover conditions can, for many individuals, prove fatal (Hewson et al. 2016; Davies et al. 2023).

Through long‐term tracking, Hewson et al. (2016) revealed UK cuckoos typically take one of two autumn migratory routes southward towards their wintering grounds in the Congo Basin. These include an eastern route via Italy, the Balkans and central Africa, and a western route via Iberia and sub‐Saharan West Africa. Compared to the eastern route, individual mortality up to completion of the Sahara crossing was significantly higher along the western route, with most of the excess mortality occurring in Europe. Moreover, there was a positive correlation between the proportion of western‐migrating cuckoos in local breeding populations with the magnitude of regional population declines (Hewson et al. 2016); specifically, western birds were more associated with lowlands of south‐east England. Thus, it is likely that conditions along the western route and/or the pre‐migratory feeding grounds are causal agents of population declines (Hewson et al. 2016; Vickery et al. 2023). While Denerley et al. (2019) and Mills et al. (2020) provide evidence that degradation of food resources has been greatest in UK lowlands, where a higher proportion of western migrants breed (Hewson et al. 2016), less is known about the conditions they (or indeed eastern birds) subsequently face on migration. Buchan et al. (2023) compared the cumulative exposure of eastern and western migrants to anthropogenic change across their annual cycle, finding elevated exposure for western birds during their wintering stage, but limited evidence for between flyway differences during autumn migration. However, their approach (which focussed on measures of anthropogenic change) does not capture potential survival threats experienced on migration which are largely independent of change (e.g., desert barriers and other inhospitable habitat; Buchan et al. 2023). As such, a knowledge gap remains regarding the environmental conditions (i.e., climate, habitat, and topography) experienced at stopovers along each route. Given that cuckoos spend 30%–40% of their annual cycle at stopover or staging sites (Willemoes et al. 2014), and quality of stopovers is known to impact the survival of other migratory species (Halupka et al. 2017; Kassara et al. 2025), elucidating conditions at these sites may greatly inform conservation efforts.

Distribution models provide a useful tool to investigate stopover use (Stevens et al. 2023). By combining telemetry and environmental data, distribution models can predict relationships between cuckoos and their stopover environment, based on preferential use of (or exposure to) environmental conditions (Elith and Leathwick 2009). If well‐built to ensure biologically realistic predictions (Guevara et al. 2018; Hazen et al. 2021), such models can also provide informative spatial projections, to identify geographic areas of potential importance on migration. However, modelling habitat use of migratory species such as the Cuckoo presents a suite of complications (Stevens et al. 2023). Firstly, migrants do not necessarily have perfect environmental knowledge of the full migratory range, potentially resulting in sub‐optimal stopover selection (Domer et al. 2021). Secondly, relationships between cuckoos and environmental variables are likely to be spatially and temporally dynamic—changing as cuckoos migrate from temperate regions in early summer through to lower‐latitude Mediterranean climates in late summer. Thirdly, the suitability of a stopover area at any given timepoint may depend on conditions in the previous months or even years. For example, habitat where contemporaneous rainfall has been sufficient to support typical abundances of invertebrate prey may in fact be unfavourable, if drought in previous years has decimated invertebrate populations (Palmer et al. 2017; Marquis et al. 2019). Furthermore, experiences at preceding stages of the annual cycle (particularly food availability at the breeding grounds; Hewson et al. 2016; Denerley et al. 2019) can influence stopover use and suitability on autumn migration (Buchan et al. 2023; Davies et al. 2023). Thus, biologically meaningful models must (a) consider cumulative effects of climatic conditions over successive seasons, (b) account for potential spatio‐temporal variation in stopover use, and (c) acknowledge that stopovers do not always necessarily reflect optimal conditions (Newton 2007; Schmaljohann et al. 2022). While the challenge of modelling migratory species is great, dramatic and continued declines of Afro‐Palearctic migrants necessitates the development of models which attempt to capture the complex environmental relationships of these species (Engler et al. 2017; Stevens et al. 2023; Vickery et al. 2023).

This study models the distribution of UK cuckoos on autumn migration along the eastern and western routes through Europe. Although autumn migratory routes extend through to sub‐Saharan Africa, this study focusses on European distribution because (a) most of the excess mortality appears to occur in Europe and (b) much of the African leg encompasses a hostile desert barrier, where comparatively few stopovers are made (Hewson et al. 2016). To address the challenges of migratory distribution modelling, this study utilises an ensemble approach, combining predictions from two machine‐learning algorithms: Boosted Regression Trees (BRTs) and Random Forests (RFs). Both algorithms have been successfully applied to understand the distribution of a wide range of migratory species (Cai et al. 2014; Zuckerberg et al. 2016; Fern and Morrison 2017; Becker et al. 2020; Kirkland et al. 2025); by integrating and comparing predictions from these two algorithms, consistency between modelling approaches can be assessed, yielding a more robust consensus of cuckoo distribution (Marmion et al. 2009). By including a suite of climatic, habitat, topographic and anthropogenic variables, this study aims to determine (1) principal environmental predictors of distribution along each route, and (2) regions of high stopover use and potential conservation priority. Doing so may prove fundamental in efforts to better understand between‐route differences in mortality rate (Hewson et al. 2016) and improve stopover suitability across the Afro‐Palearctic flyway.

## 2 Methods

### 2.1 Tracking Data

88 male cuckoos from breeding populations across the UK were fitted with Microwave Telemetry PTT‐100 tags (< 5g) between 2011 and 2021 (Hewson et al. 2016; Davies et al. 2023). The work was carried out under licence from the Special Marks Technical Panel operating on behalf of the British Trust for Ornithology and the UK Government's Home Office (Davies et al. 2023). Tags were programmed to run on a 10‐h on, 48‐h off duty cycle, thereby balancing temporal and spatial tracking resolution with the need for solar battery charging (Bonaldi et al. 2024). In addition to standard duty cycle transmissions, tags deployed after 2013 (with four exceptions) were also programmed to transmit when the battery was close to fully charged, providing extra coordinates when charging conditions were favourable (Davies et al. 2023). Occurrences were obtained via the ARGOS satellite system and subsequently filtered to the highest quality position per duty cycle (see Hewson et al. (2016) and Bonaldi et al. (2024) for further details on selection of the most accurate location).

### 2.2 Presence Data Preparation

Occurrences were first assigned an autumn migration route; individuals with migratory tracks passing through Italy or Iberia were assigned to the eastern or western route, respectively (*sensu* Davies et al. 2023). Birds whose transmitters ceased before route could be established were assumed to be following the same route as the previous year; where no data from the previous year existed, the bird was omitted from analysis (Davies et al. 2023). Occurrences were then spatio‐temporally filtered in QGIS (v.3.34.3; QGIS 2023) to those in Europe between June and August (the primary months of autumn migration; Table S2.1). UK coordinates recorded before the first movement of > 50 km (deemed the start of migration; Hewson et al. 2016) were removed, to ensure only migratory movements were included. Occurrences were also filtered to include only stopover transmissions, to prevent necessary traversal of unsuitable habitat influencing model predictions of suitable recuperation habitat. Previous estimates suggest typical stopover ranges of 5–10 km (Williams et al. 2016); however, movements within stopovers may in fact exceed these distances during migration, where birds roam more widely within suitable regions (Bonaldi et al. 2024). Stopovers were therefore defined as at least two consecutive best of duty cycle locations < 25 km apart (shorter‐term recovery periods, 3–6 days), or at least three consecutive best of duty cycle locations < 50 km apart (longer‐term refuelling and exploratory periods, > 7‐days), *sensu* Bonaldi et al. (2024). Where transmissions fell within 10 km of each other (the resolution of the coarsest environmental variable) only one location was retained, to mitigate spatial autocorrelation. Five atypical stopovers (two western‐cuckoo stopovers in Austria and three eastern‐cuckoo stopovers within/bordering Iberia) were then manually removed, to prevent distortion of the area and conditions which typically encompass the respective routes (see Appendix S1 and Figure S3.1 for how ‘atypicals’ were defined). Lastly, any coordinates missing data for one or more of the environmental variables (see below) were omitted. This resulted in 217 and 181 stopover ‘presences’ for the eastern and western routes, respectively.

### 2.3 Pseudoabsence Data Preparation

**FIGURE 1:**
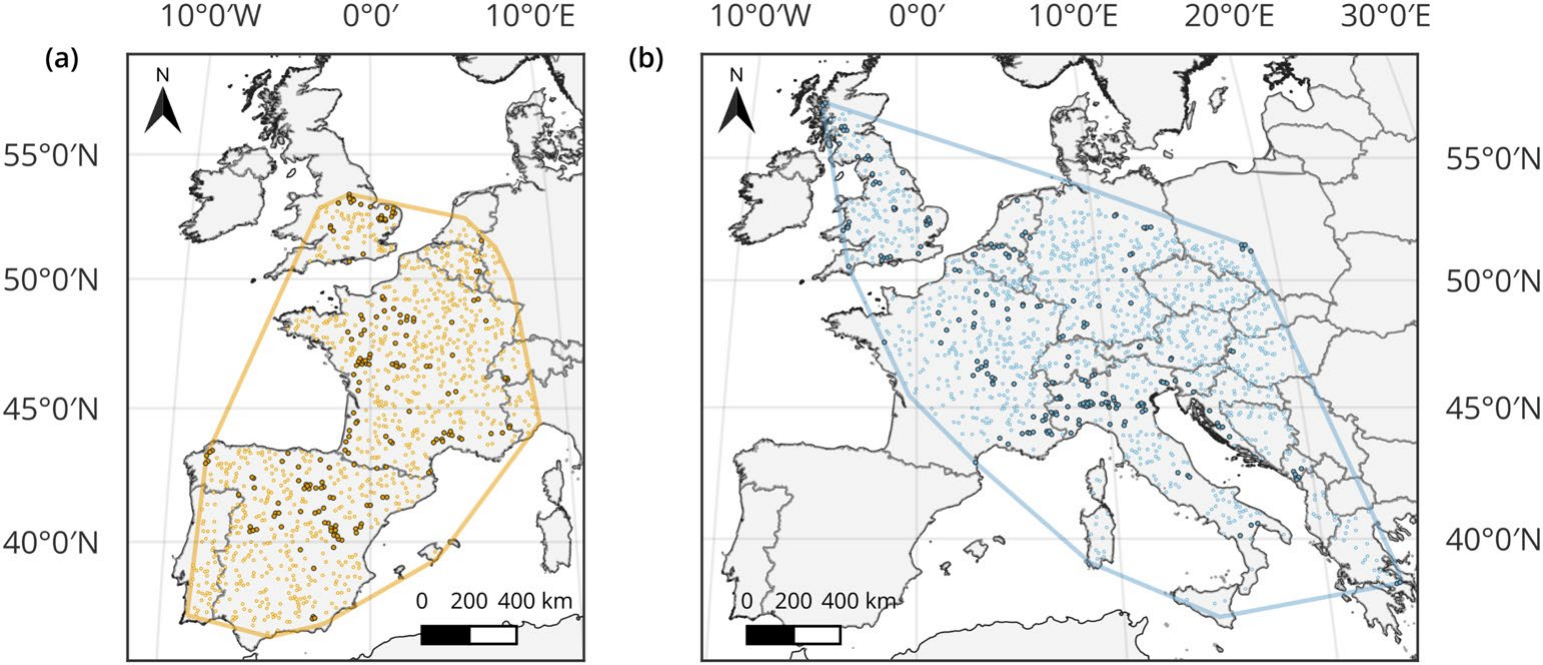
(a) filtered stopovers (orange dots) and pseudoabsences (open orange circles) within the western sampling area (orange polygon); (b) filtered stopovers (blue dots) and pseudoabsences (open blue circles) within the eastern sampling area (blue polygon).

Tracking data provides presence‐only data; thus, pseudoabsences were used to reflect locations where stopover cuckoos are likely (but not known) to be absent. To delineate the area in which eastern and western pseudoabsences were sampled, the convex hull of all eastern and western migratory movements (i.e., stopover and non‐stopover transmissions) was computed, respectively, using the R package *grDevices* (v4.1.1; R Core Team 2024). Pseudoabsences were sampled separately for each month, with pseudoabsence locations constrained to the route‐specific convex hull encompassing all tracked cuckoo movements recorded during that month. This ensured that pseudoabsences were drawn from the full area utilised by migrating cuckoos at the corresponding stage of autumn migration through Europe between 2011 and 2021 (Figure 1). A large number of pseudoabsences is often desirable to sufficiently sample the area of interest (Barbet‐Massin et al. 2012); accordingly, 4000 pseudoabsences were randomly generated for each month of each route, using the R package *sp* (v.2.1–4; Pebesma and Bivand 2005). Sampled coordinates were subsequently filtered to those > 10 km from other pseudoabsences (to mitigate spatial autocorrelation) and > 50 km from presences (to ensure pseudoabsences were not in habitat known to be used by tracked cuckoos). This resulted in a 1:5 ratio of presences to pseudoabsences (the maximum whole integer ratio achievable under the sampling constraints). However, to prevent the higher proportion of pseudoabsences (which are speculative, albeit filtered simulations) exerting greater influence on model predictions than presences (true coordinates of known stopovers), each pseudoabsence was weighted such that every five pseudoabsences carried the same weight as one presence. In this way, a sufficient number of pseudoabsences could be used to sample whole migratory areas, without eclipsing the influence of true presences. Pseudoabsences were randomly assigned a year, such that a 1:5 ratio of presences to pseudoabsences was maintained for any given year. Lastly, any coordinates missing data for one or more of the environmental variables (see below) were omitted. The resulting 1183 eastern and 931 western pseudoabsences are plotted in Figure 1.

### 2.4 Environmental Variables

Biologically meaningful models are contingent on well‐chosen, relevant variables (Fournier et al. 2017; Zarzo‐Arias et al. 2022). This requirement is particularly important for migratory species such as the Cuckoo, where variables must account for spatially and temporally dynamic environmental relationships, as well as cumulative environmental conditions over successive seasons (Stevens et al. 2023). As such, thirteen non‐correlated (|*r* |> 0.7) environmental variables with ecological relevance supported by primary literature were selected a priori (Table 1). Relevant information for each variable—including ecological justifications, resolutions and data sources—is summarised in Table 1. Note that values for each variable were extracted at their native spatial resolutions (rather than a single, harmonised resolution); resolutions ranged from ~4 × 4 km to ~10 × 10 km (Table 1), thereby aligning with typical stopover ranges, estimated at 5–10 km (Williams et al. 2016). For further details on how source data was modified to create desired variables, see Appendix S1.

**TABLE 1:** Summary of the 13 climatic, habitat, topographic and anthropogenic variables retained for modelling. Arrows indicate where the same ecological justification, data source, or resolution applies to subsequent variables.

	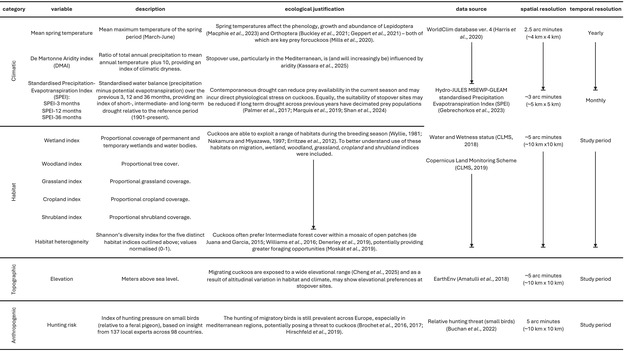

Four additional variables were also initially considered (Table S2.2), but excluded from primary modelling following collinearity analysis (Figure S3.2, Tables S2.3 and S2.4), conducted using the *vicfor* function in the *usdm* package (v.2.1–7; Naimi et al. 2014) and *cor* function from the *stats* package (v.4.4.1; R Core Team 2024). These omissions were total spring precipitation and cumulative winter precipitation (both correlated with aridity), cumulative winter temperature (correlated with mean spring temperature), and pesticide use (correlated with cropland). Selection of which correlated variable to retain for primary models was based on supporting literature (see Appendix S1); however, to assess the impact of alternative variable selection on model outputs, we conducted a supplementary sensitivity analysis by repeating our modelling approach (see below) while substituting each variable in a correlated pair.

### 2.5 Modelling

#### 2.5.1 Model Selection

In this study, Boosted Regression Tree (BRT) and Random Forest (RF) models were employed, before combining their predictions to create weighted ensemble models for each route. Both algorithms can model non‐linear environmental relationships and interactions between variables, making them well‐suited to modelling migratory distributions (Cai et al. 2014; Zuckerberg et al. 2016; Fern and Morrison 2017; Becker et al. 2020; Kirkland et al. 2025). Furthermore, both BRTs and RFs can handle a large number of predictor variables, being robust to the inclusion of variables which may ultimately contribute little to model predictions (Cutler et al. 2007; Hastie et al. 2009; Evans et al. 2010; Elith and Leathwick 2017). This dual‐model approach allows consistency between algorithms to be assessed, improving confidence in predicted environmental relationships and spatial distributions (Marmion et al. 2009). A third, widely used algorithm in distribution modelling—MaxEnt (Elith et al. 2011)—was also trialled (see Appendix S1 ‘MaxEnt model tuning and fitting’ and accompanying Tables S2.5–S2.7), but dropped after prior testing showed it underperformed tree‐based models (Table S2.7).

#### 2.5.2 Boosted Regression Tree Models

For both routes, BRTs were fit with the *gbm.step* function from the *dismo* package (v.1.3–14; Hijmans et al. 2023). The *gbm.step* function uses 10‐fold cross validation to estimate the optimal number of trees (Elith and Leathwick 2017); in this case, 8000 for the eastern model and 8450 for the western model. Models were fit with a Bernoulli distribution. Tree complexity was set to four, allowing up to four‐way interactions between variables. It should be noted that 5‐fold cross validation model tuning (using the *train* function from the *caret* package, v.6.0–94; Kuhn 2008) suggested higher tree complexities (> 10) yielded marginally higher AUC values. However, concerned that such high‐order interactions may reflect overfitting to our small dataset, we considered up to 4‐way interactions to provide appropriate balance between predictive performance and ecologically realistic environmental relationships scalable to the wider population. The learning rate (shrinkage factor for each individual regression tree) was set to 0.001; such low values require many trees but as a result, typically provide more robust predictions (Elith et al. 2008). The bag fraction was set to 0.5—a value commonly used to introduce an appropriate degree of stochasticity to the model, which generally improves accuracy and prevents overfitting (Friedman 2002; Elith et al. 2008). In addition to the thirteen environmental variables considered in this study (Table 1), month and year were also included as predictors. These variables were not expected to have substantial fixed effects, but could potentially interact with the thirteen primary variables, reflecting temporal trends in environmental relationships.

#### 2.5.3 Random Forest Models

Random forest models were fit using the *randomForest* function from the *randomForest* package (v.4.7–1.2; Liaw and Wiener 2002). The number of variables considered at each split was set as 4 for both routes (*sensu* BRT models described above). All remaining parameters were set to default, following Probst et al. (2019) and Valavi et al. (2021). As with BRTs, month and year were included alongside environmental variables.

#### 2.5.4 Ensemble Models

Ensemble models for each route were built by combining the predictions of RFs and BRTs, weighting their influence by respective True Skill Statistic (TSS) and Area Under the Receiver Operator Curve (AUC) values (see the following section on ‘Model Evaluation and Interpretation’).

Finally, we repeated the above modelling approach with the addition of latitude as a predictor variable. By allowing environmental variables to interact with latitude and detect potential latitudinal trends, models may be able to generate more accurate spatial predictions of stopover distribution. However, latitude may also exert strong explanatory power due simply to its ability to describe the spatial distribution of stopovers (see ‘Results’ and 'Discussion'), potentially obscuring the underlying environmental relationships which drive latitudinal preferences. Hence, latitude was excluded in one set of models to determine the predictive power of environmental variables alone.

### 2.6 Model Evaluation and Interpretation

Models were evaluated using 10‐fold cross‐validation, whereby data was randomly divided into ten subsets (folds); each fold served as a testing set once, while the remaining nine folds formed the training set. This approach ensured that all data contributed to both model training and independent evaluation. Moreover, averaging evaluation metrics across ten iterations provides a more robust measure of model performance (Vabalas et al. 2019).

Predictive skill was evaluated using the mean TSS and AUC of each model, both of which measure the ability of models to distinguish presences from pseudoabsences (Valavi et al. 2021). TSS is a threshold‐dependent metric and was here calculated using the maximum training sensitivity plus specificity (MTSS) threshold, where sensitivity is the proportion of correctly predicted presences and specificity is the proportion of correctly predicted pseudoabsences (Liu et al. 2013). The MTSS threshold balances the risk of misclassifying either presences or pseudoabsences, offering a single objective classification threshold well suited to presence‐only data with randomly generated pseudoabsences (Liu et al. 2013). TSS values range from −1 (worse than random) to 1 (perfect prediction). AUC is threshold‐independent, with values ranging from 0.5 (no better than random) to 1 (perfect distinction). While acknowledging the potential shortfalls of the AUC as a measure of model fit (Lobo et al. 2008), its use here is justified on the grounds that (a) its prevalence in the literature makes it highly interpretable, scrutable, and comparable with other modelling studies, and (b) it is supplemented with additional evaluative approaches.

Variable importance was assessed using the mean permutation percentage decrease in accuracy, calculated using the *vi* function from the *vip* package (v.0.4.1; Greenwell and Boehmke 2020). Relationships between cuckoos and their stopover environment were determined by examining partial response curves for each variable. Important stopover sites were identified by inspecting suitability maps generated by model predictions. Predictions were made for the highest and lowest drought years based on the SPEI3 variable (Table 1), thereby capturing potential effects of recent drought (over the previous 3 months) on stopover distribution (Hewson et al. 2016). For the eastern route, drought coverage was lowest during 2014 and greatest during 2015, while for the western route it was lowest during 2021 and highest during 2015 (see Appendix S1 and Figures S3.3–S3.6 for how annual drought coverage was determined). For each of these years, a seasonal prediction was attained by averaging monthly predictions. Finally, predictions for each year were converted to binary plots, using the mean MTSS threshold from 10‐fold cross validation. Change maps were then plotted to assess variation in predicted stopover probability between high and low drought years.

## 3 Results

### 3.1 Evaluation Metrics

Predictive ability was moderately high across model‐types and migration routes, with mean AUC and TSS values ranging from 0.79–0.88 and 0.50–0.66, respectively (Table 2). For both eastern and western routes, RF and ensemble models yielded marginally higher AUC and TSS values than BRTs (Table 2), indicating slightly superior predictive performance. Across all algorithms and migration routes, the inclusion of latitude as a predictor variable enhanced AUC values by 0.01–0.03 and TSS values by 0.0–0.07 (Table 2), suggesting predictive performance is only marginally enhanced when latitudinal trends in stopover distribution are explicitly modelled.

**TABLE 2:** Area Under the Receiver Operating Characteristic Curve (AUC), True Skill Statistic (TSS), and maximum training sensitivity plus specificity (MTSS) threshold for all models. Values are the mean from 10‐fold cross validation, with 95% confidence intervals in parentheses. Dashed lines separate models by migration route and the inclusion of latitude (‘+lat’); for each group of models, the highest AUC and TSS values are in bold.

	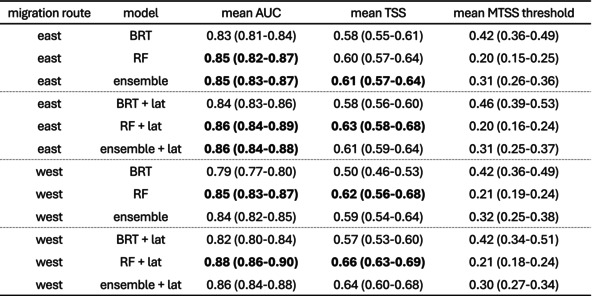

Sensitivity analyses (where total spring precipitation, cumulative winter precipitation, cumulative winter temperature and pesticide use were substituted for aridity, mean spring temperature and cropland) revealed minimal changes in model performance, variable rankings, environmental relationships or spatial predictions, indicating model outputs outlined below remain robust when alternative variables are selected. For full details, see Appendix S1 ‘sensitivity analysis results’ and accompanying Table S2.8, Figures S3.9, S3.14, S3.21 and S3.22.

### 3.2 Variable Importance and Environmental Relationships

**FIGURE 2:**
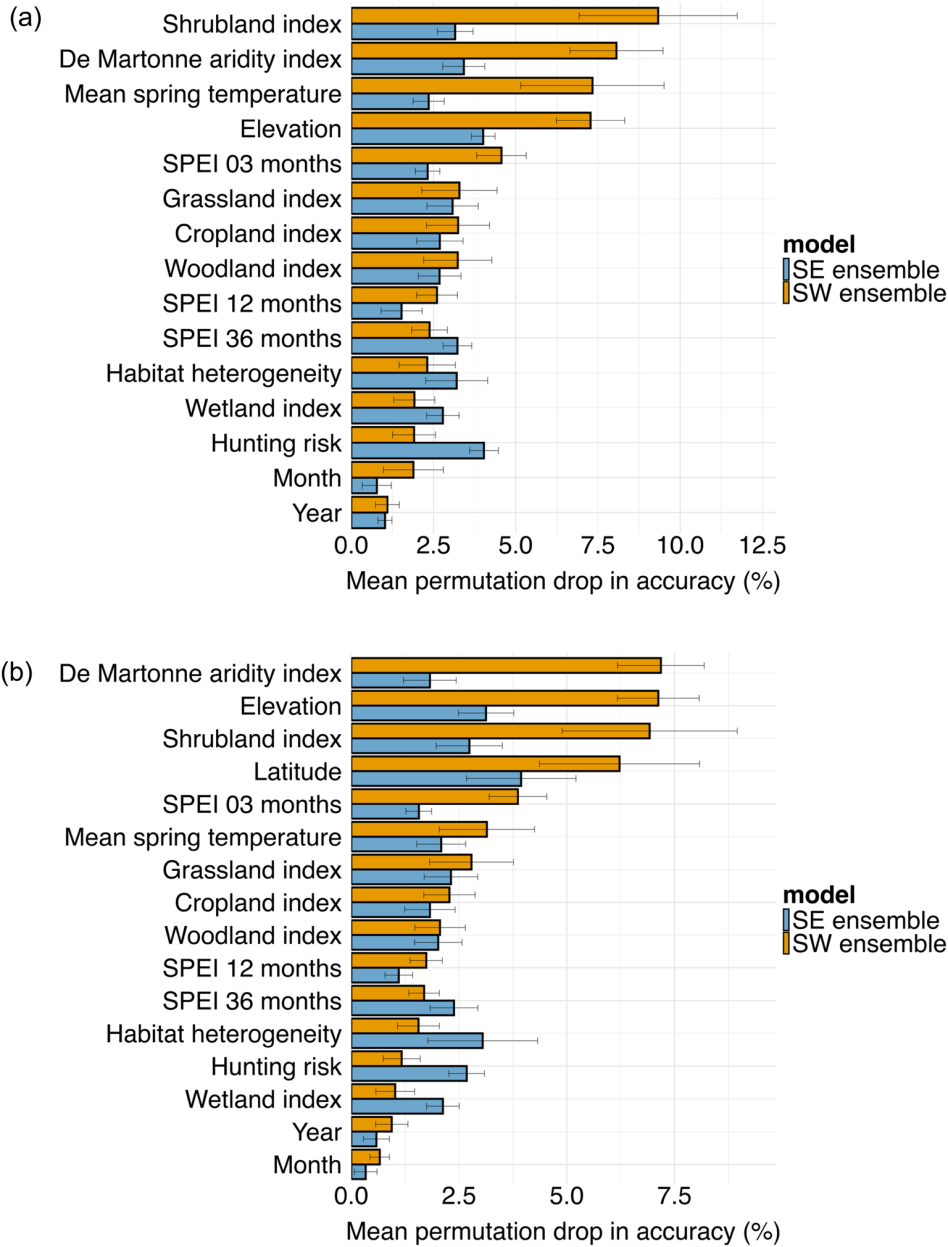
Percentage drop in accuracy following permutation of each variable for (a) ensemble models excluding latitude, and (b) ensemble models including latitude as a predictor. Values represent the mean from 10‐fold cross validation, with whiskers indicating upper and lower 95% confidence intervals. Variables are ranked in descending order for the western route, with their comparative importance for the eastern route.

Permutation analyses revealed between‐route differences in the key predictors of stopover distribution (Figure 2). For the western route, shrubland cover, De Martonne Aridity index (DMAI), mean spring temperature, elevation, and SPEI3 were consistently the strongest predictor variables across ensemble, RF, and BRT models (Figures 2 and S3.7). For the eastern route, hunting risk, elevation, DMAI, SPEI36, and habitat heterogeneity were the highest‐ranking variables for ensemble models (Figure 2). Constituent RF and BRT models had slight discrepancies on variable rankings, with some intermediate ranking variables (including grassland, DMAI, and shrubland) ranked higher for RFs than BRTs (Figure S3.7); nonetheless, eastern ensemble rankings represent the consensus between constituent models, reflecting the strongest and most consistent signals identified across both algorithms (Figure 2).

Across routes, habitat predictors were generally of intermediate‐to‐low importance (except shrubland and habitat heterogeneity), whereas climatic, topographic, and anthropogenic factors were more predictive. Temporal variables (year, month) consistently ranked lowest. When included, latitude emerged as one of the strongest predictors for both routes (Figures 2 and S3.8), yet did not substantially improve overall predictive performance (Table 2), indicating redundancy in its effect when other environmental variables are considered.

**FIGURE 3:**
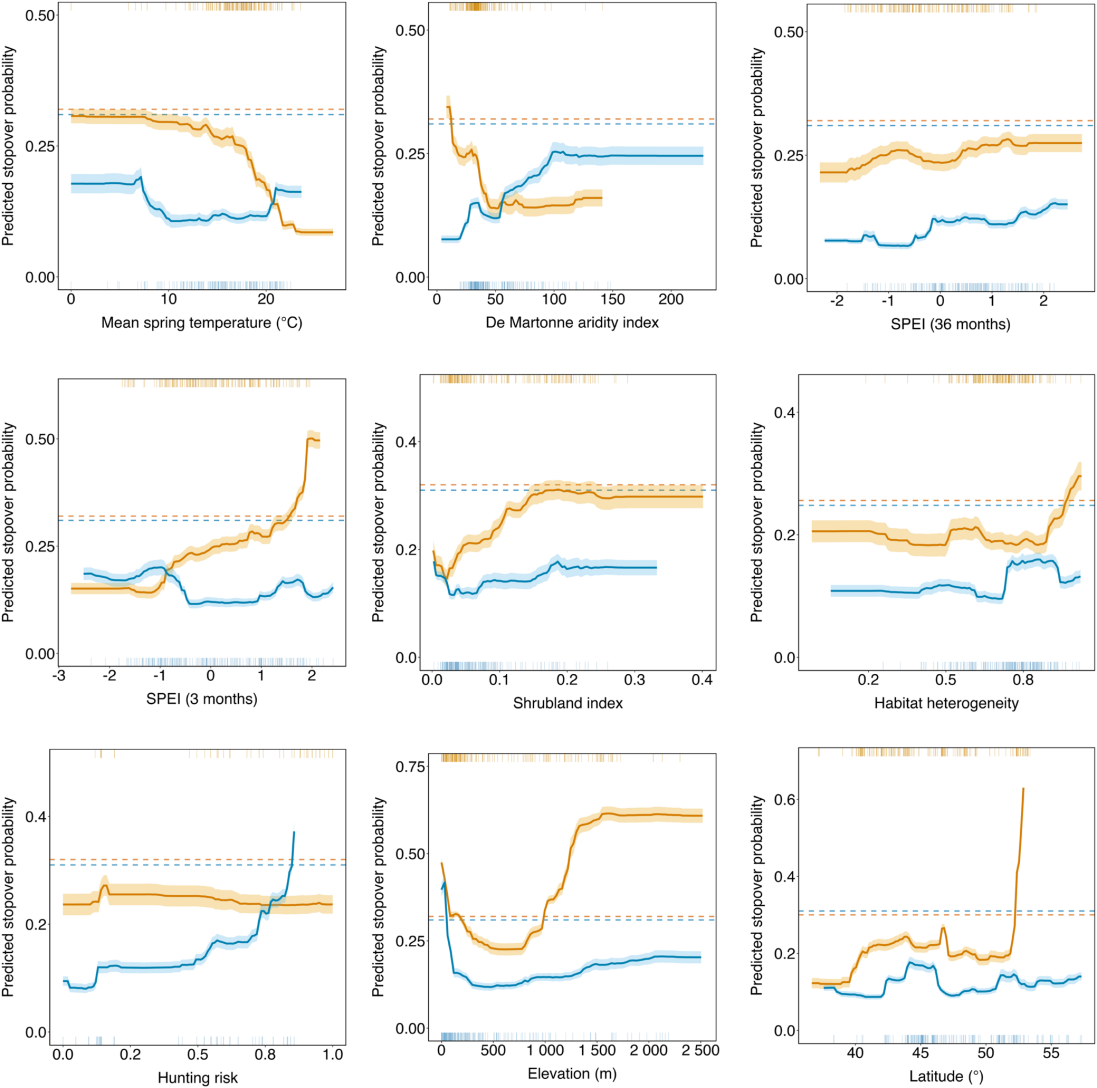
Response curves (with 95% confidence intervals) for the eastern (blue) and western (orange) ensemble models, with latitude included as a predictor. Included are the top five environmental predictors for the eastern route (hunting risk, habitat heterogeneity, elevation, aridity and SPEI36) and western route (aridity index, elevation, spring temperature, SPEI3 and shrubland), as well as latitude. Blue and orange dashed lines indicate the mean max TSS threshold for eastern and western models, respectively. Rugs denote true stopovers.

Response curves from ensemble models (Figures 3, S3.10 and S3.11) depict environmental relationships broadly consistent across all model types (Figures S3.12 and S3.13). Along the western route, stopover probability was highest in semi‐arid to semi‐humid regions (DMAI values < 35), dropping sharply in more humid regions and above ~18°C mean spring temperature (Figure 3). Stopover probability was also highest in lowland (< 100 m) and montane (> 800 m) regions with intermediate shrubland cover and low drought in recent months (SPEI3 values > 1). With latitude included, probability was highest at latitudes > 52° N: the breeding grounds. Response curves also depict a less pronounced peak in stopover probability between 42° and 49° N, before dropping below 40° N (Figure 3). For the eastern route, stopover probability was greatest in more humid areas of high habitat heterogeneity and high hunting risk, again peaking in lowlands and increasing in montane regions (Figure 3). When latitude was included, response curves show a modest peak in stopover probability around 45^o^N. Response curves for lower‐ranking variables and constituent models are provided in Appendix S3 (Figures S3.10–S3.13).

### 3.3 Spatial Predictions

**FIGURE 4:**
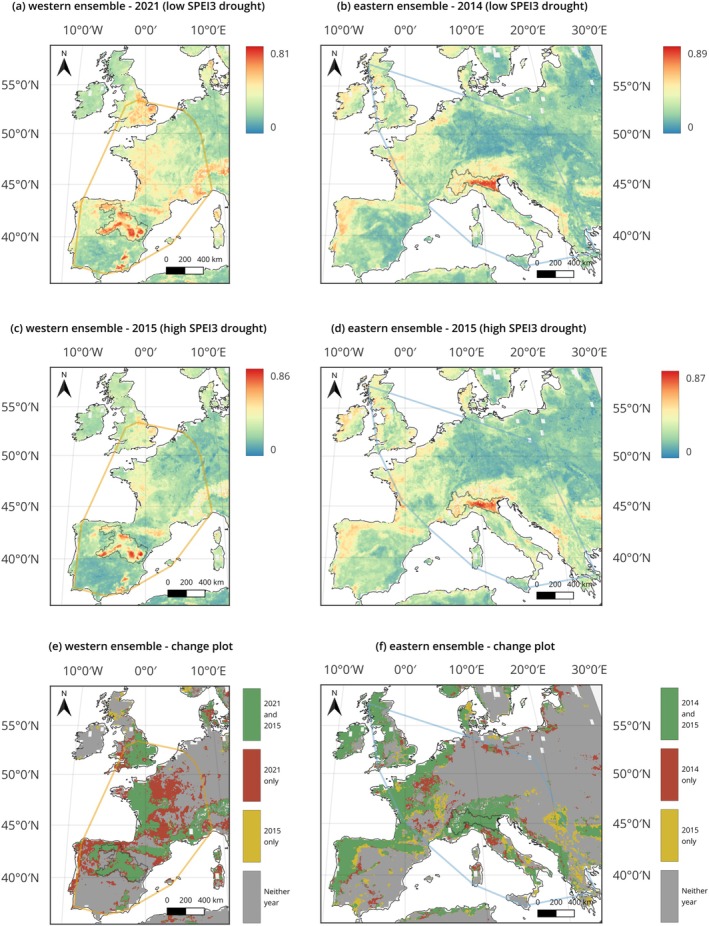
Ensemble spatial predictions for the western (a, c, e) and eastern (b, d, f) migration routes. Heat maps for years of low (a & b) and high (c & d) SPEI3 drought indicate stopover probability (see keys). Key regions of predicted stopover use are outlined by black borders. For change maps (e & f), colours indicate whether stopovers were predicted across one, both or neither years (see keys). Polygons denote the area in which models were trained. Maps are projected using the Mollweide projection (ESRI: 54009).

**FIGURE 5:**
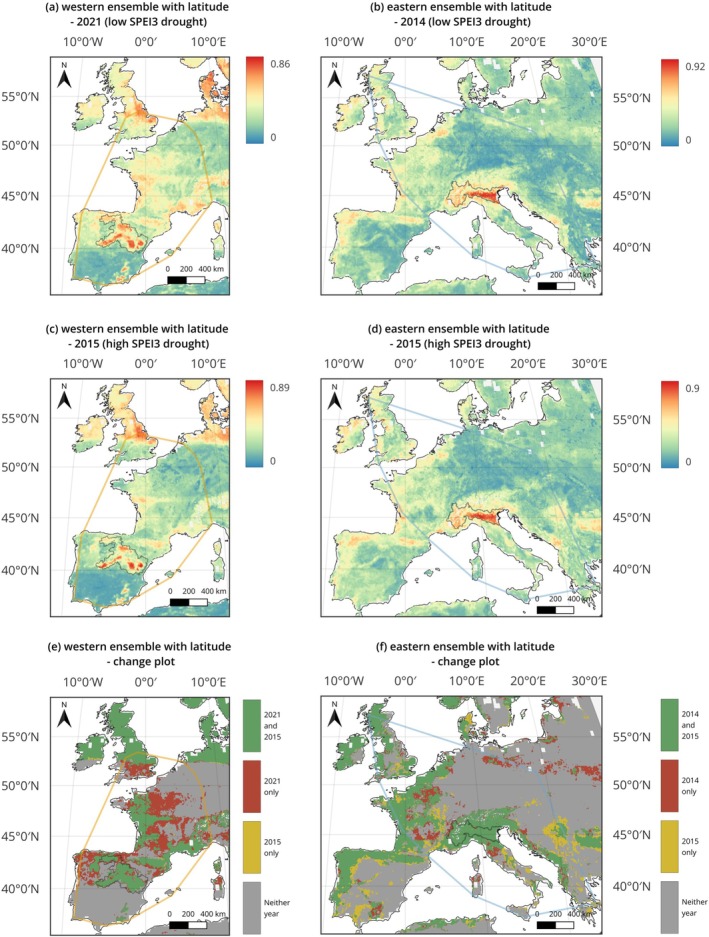
Ensemble spatial predictions for the western (a, c, e) and eastern (b, d, f) migration routes, with latitude included as a predictor. Heat maps for years of low (a & b) and high (c & d) SPEI3 drought indicate stopover probability (see keys). Key regions of predicted stopover use are outlined by black borders. For change maps (e & f), colours indicate whether stopovers were predicted across one, both or neither years (see keys). Polygons denote the area in which models were trained. Maps are projected using the Mollweide projection (ESRI: 54009).

For both routes, RF models yielded more conservative predictions of probable stopover sites than BRTs (Figures S3.15–S3.18); nonetheless, both algorithms highlighted the same key areas of high stopover probability visible in ensemble predictions (Figure 4). Western predictions highlight a large band of probable stopovers in Northwest and central France, largely within the Loire and Seine River basins. As cuckoos migrate southward towards Iberia, stopovers are predicted at the eastern and western edges of the Pyrenees, along the ‘Atlantic and Mediterranean Corridors’ (Galarza and Tellería 2003; Fourcade et al. 2022). In Spain, predictions followed a clear pattern of high probability around the major mountain systems, particularly the Sistema Ibérico, Sistema Central, Cordillera Cantábrica, and Baetic mountains; stopover probability in the semi‐arid expanses surrounding these mountain systems was low. The inclusion of latitude as a predictor variable resulted in a strong increase in probable stopover sites above 52^o^N (Figure 5); below 52° N, predicted stopover distribution differed little from models without latitude (Figure 4). The spatial extent of key stopover regions—and the probability of them being utilised—were reduced during 2015 (the year of highest SPEI3 drought) compared to 2021 (low drought; Figures 4 and 5).

For the eastern route, spatial predictions highlight the River Po watershed as a key stopover site, with areas of central France and Benelux of lesser importance (Figure 4). Some stopover sites were also predicted in regions of Slovenia, Croatia, and along the Adriatic coast. The inclusion of latitude did not appear to substantially alter spatial predictions (Figure 5). Predictions also showed little change between 2014 (low SPEI3 drought) and 2015 (high SPEI3 drought); however, SPEI3 exerted only a minor influence on eastern stopover probability (Figure 2). Comparison of spatial predictions for 2014 and 2019 (the years of low and high SPEI36 drought, respectively) shows a more substantial reduction in the probability and extent of stopover sites (Figures S3.19 and S3.20).

## 4 Discussion

Distribution models are valuable tools for understanding species‐environment relationships and the distributions of avian migrants, provided both the study system and modelling approach are rigorously considered and limitations acknowledged (Engler et al. 2017; Stevens et al. 2023). Accordingly, this study combines biologically relevant variables with two alternative algorithms to create ensemble models for two autumn migration routes of UK cuckoos. For each route, algorithms converged on (a) the most influential predictors of distribution, (b) environmental relationships, and (c) key stopover sites, providing greater confidence in the validity of these findings, which reveal notable between‐route differences in environmental drivers of stopover distribution.

### 4.1 Eastern Route

The strongest environmental predictor for the eastern route was hunting risk, revealing a tendency for birds to stop in high‐threat areas. It should be noted that the hunting risk variable used in this study encompasses risk to all small migratory birds and does not distinguish between legal and illegal hunting pressures (Buchan et al. 2022); thus, it is hard to discern the direct threat posed to cuckoos specifically. However, much of the legal hunting at sites along the eastern route concentrates on larger quarry species (mostly wildfowl) which are not included in the hunting risk variable used here; therefore, the apparent risk to eastern cuckoos is not simply driven by the threat of legal wildfowl hunting. Both anecdotal and photographic evidence of cuckoos being shot or caught in mist nets along the eastern route (Axel Hirschfeld, pers. com.), as well as the pervasiveness of illegal poaching across the Mediterranean (Brochet et al. 2016, 2017; Hirschfeld et al. 2019), suggest cuckoos are certainly under some degree of risk. Whatever the extent of illegal hunting, it constitutes an additional and unnecessary hazard which may compound other environmental stressors experienced on route. Enhancing international coordination to tackle illegal hunting will therefore be essential to mitigate direct anthropogenic threats to migratory birds (Barca et al. 2016; Vickery et al. 2023; Sánchez‐García et al. 2024).

Spatial predictions identify the Po Watershed as a key stopover region. On field visits to this site, we observed heavy use by cuckoos of the valley floor, little use of densely forested intermediate elevations, and higher use of upland mosaics maintained by natural disturbance (C.M.H., pers. obs.). These observations are consistent with response curves illustrating a preference for heterogeneous habitat at low and high elevations—corroborating previous studies also detecting a preference for semi‐natural mosaics (Williams et al. 2016; Denerley et al. 2019). Despite its importance as a stopover site, the Po Valley faces considerable environmental pressures; much of this agricultural region is ecologically degraded (Provini and Binelli 2005; Barca et al. 2016; Corbau et al. 2019) and is considered high‐risk for Lepidopteran populations due to rapidly increasing temperatures and nitrogen deposition (Rashid et al. 2023). Additionally, our sensitivity analyses indicate eastern birds are more likely to stop in agricultural areas with higher quantities of pesticide application (Figure S3.14), further threatening insect prey populations at key stopover sites (Rigal et al. 2023). Notwithstanding concerns over the vulnerability of prey to anthropogenic climate change (ACC) and agricultural intensity in this region (Rashid et al. 2023), it is perhaps encouraging that cuckoos can utilise anthropogenic landscapes on migration. Combined with an apparent preference for mosaic habitat, this study emphasises the importance of including semi‐natural habitat within agricultural land (IUCN 2023) to safeguard this key stopover site.

Spatial predictions did not depict a substantial change in stopover distribution between years of low (2014) and high (2015) SPEI3 drought (Figures 4 and 5). However, none of the SPEI variables emerged as primary predictors (SPEI36 was the highest ranking of the three; Figure 2), indicating models were unable to use drought variables to effectively distinguish presences from pseudoabsences. Indeed, drought conditions occurred at both presence and pseudoabsence locations during 2015 (Figure S3.3), likely limiting the ability of drought metrics to discriminate between classes. However, this does not mean eastern birds are unaffected by drought. Certainly, we observed behavioural changes in birds attempting to stop in the Po watershed during severe 2015 droughts (C.M.H., pers. obs.), followed by multiple deaths along the eastern route that year. Furthermore, spatial predictions for 2019—the year of highest SPEI36 drought—do show a reduction in the extent and probability of stopover sites (Figures S3.19 and S3.20), suggesting that while short‐term drought may alter behaviour without reshaping broad stopover distributions, prolonged or cumulative drought may ultimately reduce the availability and reliability of stopover habitat (Londe et al. 2025). Thus, while other environmental variables have proved stronger predictors of stopover distribution in this study, drought and other extreme climatic events are still likely to impact the suitability of these sites. Finer‐scale GPS data and behavioural studies within key stopover regions may be better able to detect sensitivity to such extreme climatic events (Bayly et al. 2018; Ramos et al. 2023; Naves‐Alegre et al. 2025) and are therefore recommended as a potentially fruitful avenue of future research.

### 4.2 Western Route

Western stopover probability was high in semi‐humid areas at higher elevations, where mean spring temperatures did not exceed 18°C; accordingly, the Iberian mountain ranges emerged as key stopover areas. Together, these environmental relationships and spatial predictions suggest a preference for mesic habitat in the supra‐Mediterranean climatic belt (typified by a moderate supply of moisture, cooler temperatures, and higher altitudes)—consistent with the bioclimatic preferences of Iberian breeding cuckoos (de Juana and Garcia 2015). Given this apparent preference for mesic habitat, projected increases in the intensity and persistence of arid conditions across Iberia (Andrade et al. 2021; Arias et al. 2021) could reduce the suitability of sites along the western route. Indeed, aridity‐associated reductions in food availability at Iberian stopover sites have already been attributed to reduced survival in several other insectivorous Afro‐Palearctic migrants (Halupka et al. 2017; Goffin et al. 2020; Kassara et al. 2025). Furthermore, as the rate of warming increases with elevation (‘elevation‐dependent warming’; Pepin et al. 2015), precipitation at higher altitudes is also projected to decrease (Carrillo et al. 2023); thus, increasing aridity is likely to be more pronounced at higher altitudes (Carvalho et al. 2022), where cuckoos appear to be stopping on migration (Figures 4 and 5). As such, these findings underscore the importance of mitigating Mediterranean desertification under ACC (Arias et al. 2021).

While aridity in this study indicates climatic dryness, the SPEI measures the intensity of ground water deficit/surplus across various time scales, reflecting the intensity of drought (Table 1). For the western route, SPEI3 was the most predictive drought variable, with response curves indicating a strong preference for sites of low drought in the three months prior to stopping (Figure 3). Given a previous hypothesis that mortalities along the western route may have been partly driven by drought (Hewson et al. 2016), it is encouraging that western stopover probability was greatest in low (short‐term) drought areas, and suggests cuckoos are able to track favourable climatic conditions during migration (Thorup et al. 2017). Nevertheless, increasingly frequent and severe droughts across Iberia (Chidean et al. 2025) are likely to inhibit the ability of cuckoos to locate low drought areas. Indeed, spatial predictions show a reduction in the extent and probability of stopover sites during 2015—the year when SPEI3 drought was highest during the migratory period (Figure 4). Reduced prey populations (Klockmann and Fischer 2017; Herrando et al. 2019) and/or direct physiological stress during drought conditions are likely to jeopardise the suitability of key stopover sites, raising concerns over the viability of Afro‐Palearctic migratory routes during drought events.

Despite the vulnerability of high elevation Iberian stopover sites to ACC (Andrade et al. 2021; Carvalho et al. 2022; Carrillo et al. 2023), several characteristics of these mountain ranges make them excellent locations to buffer climatic change and enhance habitat suitability. Topographic heterogeneity at higher altitudes can provide macro‐ and micro‐climatic refuge (such as shade) from more hostile regional conditions (Correia et al. 2015; Goded et al. 2024; Ursul et al. 2024), and often precludes more intensive anthropogenic land uses, providing an excellent opportunity to prioritise conservation (Triviño et al. 2018; Ursul et al. 2024). In addition, the Iberian Mountain ranges are reasonably well covered by Protected Area (PA) designations and well poised for research‐informed expansions and regulatory enhancements (Romo et al. 2023). Given that UK cuckoos are poorly represented by PAs across their migratory cycle (Border et al. 2025), and the apparent importance of Iberian montane regions as stopover sites, this study echoes calls from the published literature (Triviño et al. 2018; Romo et al. 2023) for focussed conservation efforts in these regions. While the efficiency of static area‐based conservation measures can be confounded by the highly mobile, wide‐ranging nature of migratory species (Runge et al. 2014), cuckoos demonstrate fidelity for sites where they have previously made prolonged stopovers (Bonaldi et al. 2024). Thus, targeted conservation at frequently used stopover sites—such as those in the Iberian Mountain systems and the Po watershed—could significantly enhance cuckoo survival (Runge et al. 2014; Triviño et al. 2018).

### 4.3 Latitudinal Trends and Their Effect on Predicted Stopover Distribution

When included, latitude emerged as one of the strongest predictors for both routes (Figure 2), incurring a marginal increase in predictive performance (Table 2). However, its inclusion did not substantially alter variable rankings, response curves, or predicted distribution. Thus, the additional explanatory power of latitude does not change the key inferences made from models built using environmental variables alone. The most parsimonious explanation for latitude's predictive power is that it is largely redundant, capturing spatial patterns already explained by environmental variables. The small improvement in predictive performance likely reflects residual spatial structure detected by latitude, potentially arising from idiosyncratic site selection of individuals, interactions between latitude and environmental variables, or unmeasured environmental factors proxied by latitude. Perhaps the most noteworthy point raised by the inclusion of latitude is the sharp spike in predicted probability along the western route at ~52^o^N: southeast England breeding grounds. Because stopovers were filtered to exclude transmissions prior to the first movement > 50 km (*sensu* Hewson et al. 2016), this pattern suggests either (a) breeding‐ground home ranges exceed 50 km, or (b) having initiated migration, western birds stop over closer to the breeding grounds. Larger home ranges would be consistent with Williams et al.'s (2016) suggestion that cuckoos occupy more extensive areas of lower‐quality habitat (such as agriculturally dominated lowland Britain; Denerley et al. 2019) in order to attain sufficient resources, while short initial migratory hops may reflect insufficient fuelling, owing to reduced caterpillar abundance in Southeast England (Denerley et al. 2019; Fox et al. 2021). In either case, the inference is that birds breeding in lowland Britain—particularly western migrants—are inhabiting lower‐quality habitat.

### 4.4 Drivers of Distribution at a Flyway‐Scale

Notwithstanding the high‐ranking importance of habitat heterogeneity and shrubland cover (Figure 2), climatic (aridity, spring temperature and drought), topographic (elevation) and anthropogenic (hunting risk) features consistently emerged as the most influential predictors for both routes. Distribution models for other Afro‐Palearctic migrants yield similar findings; suitable habitat for the Nightingale ( *Luscinia megarhynchos* ) at its non‐breeding grounds in tropical Africa is largely predicted by elevation, drought and precipitation (Kirkland et al. 2025), and climatic variables exert the greatest influence on predicted distribution of the European Bee‐eater ( *Merops apiaster* ; Abdul‐Wahab et al. 2024). Such studies (including this one) often use relatively coarse resolution models to effectively encompass the extensive ranges of these highly mobile species (Pocewicz et al. 2013; Buderman et al. 2016; Coxen et al. 2017; Engler et al. 2017). At such scales, climatic and topographic gradients are typically steeper than those of habitat variables (Field et al. 2008); thus, the former often prove stronger predictors of presence and absence across wider spatial extents (Guisan and Thuiller 2005; Austin and Van Niel 2011; Estrada‐Villegas et al. 2012). Consequently, coarse‐resolution models can conceal habitat preferences (Austin and Van Niel 2011), perhaps explaining the low‐to‐intermediate importance rankings of some habitat variables in this study. Certainly, finer‐scale studies focussing on the home ranges of breeding cuckoos have detected habitat preferences (Campobello and Sealy 2009; Williams et al. 2016; Moskát et al. 2019), underscoring the critical role of spatial scale in identifying drivers of distribution (McGill 2010; Gillingham et al. 2012). The results of this study suggest climatic and topographic features largely determine the distribution of suitable stopover areas at a flyway‐scale, identifying broad areas of importance to migratory cuckoos. While also detecting a preference for heterogeneous habitat along the eastern route, additional habitat preferences may be elucidated by finer scale models (Engler et al. 2017; Crispim‐Mendes et al. 2024) – a direction of research becoming increasingly viable with the advent of GPS trackers (Lathouwers et al. 2023) recently deployed on UK and Irish cuckoos. The miniaturisation of GPS trackers also brings potential for females and juveniles (which are generally smaller than males) to be tagged, providing future opportunity to assess the generality of our findings to the entire cuckoo population.

## Author Contributions

**Alex J. Gale:** conceptualization (equal), data curation (equal). **Máire Kirkland:** conceptualization (equal), data curation (equal). **Philip W. Atkinson:** conceptualization (equal), data curation (equal). **Robert Puschendorf:** conceptualization (equal), data curation (equal). **Chris M. Hewson:** conceptualization (equal), data curation (equal).

## Funding

This study and the BTO Cuckoo Tracking Project were funded by the A. G. Leventis Foundation, BBC Wildlife Fund, Dulverton Trust, BTO including from much appreciated gifts in Wills, Ernest Kleinwort Charitable Trust, Essex and Suffolk Water, Mark Constantine, Tobit Trust and ‘Cuckoo Sponsors and Champions’ (including Anglian Water, the Broads Authority, Dartmoor National Park, Devon Birds, Gillian & Justin Wills, Severn Trent Water and Sussex Ornithological Society).

## Ethics Statement

The tagging was licenced by the Special Marks Technical Panel operating on behalf of the British Trust for Ornithology and the UK Government's Home Office and carried out in accordance with their ethical guidelines.

## Conflicts of Interest

The authors declare no conflicts of interest.

## Supporting information




**Appendix S1:** ece374083‐sup‐0001‐Appendix S1.docx.

**Appendix S2:** ece374083‐sup‐0002‐Appendix S2.pdf.

**Appendix S3:** ece374083‐sup‐0003‐Appendix S3.pdf.

**Data S1:** ece374083‐sup‐0004‐supinfo.csv.

**Data S2:** ece374083‐sup‐0005‐supinfo.R.

**Data S3:** ece374083‐sup‐0006‐supinfo.R.

**Data S4:** ece374083‐sup‐0007‐supinfo.R.

## Data Availability

All environmental data used in this study is freely accessible from public repositories detailed in the article, supporting information and references. The tracking data and code supporting our analyses have been provided in the Supporting Information. Access to underlying tracking data is subject to the data sharing policies of the British Trust for Ornithology.
