# Supplementary material for: Drivers of Stopover Distribution Across Two Autumn Migration Routes of a Long‐Distance Afro‐Palearctic Migrant, the Common Cuckoo ( Cuculus canorus )
Source: Ecol Evol. 2026 Aug 4;16(8):e74083. doi: 10.1002/ece3.74083 (PMC13435883; doi:10.1002/ece3.74083)
Supplement: Supplementary file 1 [file ECE3-16-e74083-s001.pdf]

Appendix S2

Table S2.1: total occurrences in Europe for each month and year. Rows in bold denote the primary months of autumn migration through Europe, and the occurrences taken forward for further analysis. Transmissions in the months prior to June depict local movements within the breeding range; low transmissions numbers in Europe beyond August reflect arrival in Africa, mortalities and failed transmissions. Where months are missing for a given year, no satellite transmissions were received within Europe for that month.

Year	Month	Total occurrences
2011	May	17
**2011**	**June**	**60**
**2011**	**July**	**32**
**2011**	**August**	**4**
2012	April	12
2012	May	93
**2012**	**June**	**181**
**2012**	**July**	**155**
**2012**	**August**	**47**
2012	September	15
2012	October	1
2013	April	34
2013	May	157
**2013**	**June**	**268**
**2013**	**July**	**192**
**2013**	**August**	**42**
2013	September	1
2014	April	45
2014	May	205
**2014**	**June**	**385**
**2014**	**July**	**220**
**2014**	**August**	**47**
2014	September	5
2015	April	60
2015	May	179
**2015**	**June**	**271**
**2015**	**July**	**129**
**2015**	**August**	**31**
2015	September	9
2016	April	41
2016	May	59
**2016**	**June**	**153**
**2016**	**July**	**146**
**2016**	**August**	**49**
2016	September	14
2016	October	1
2017	March	3
2017	April	37
2017	May	104
**2017**	**June**	**140**
**2017**	**July**	**59**
**2017**	**August**	**21**
2017	September	12
2017	October	10
2018	April	33
2018	May	131
**2018**	**June**	**205**
**2018**	**July**	**107**
**2018**	**August**	**1**
2019	April	24
2019	May	134
**2019**	**June**	**146**
**2019**	**July**	**87**
**2019**	**August**	**2**
2020	April	19
2020	May	55
**2020**	**June**	**42**
**2020**	**July**	**35**
**2020**	**August**	**21**
2020	September	1
2021	April	8
2021	May	78
**2021**	**June**	**181**
**2021**	**July**	**90**
**2021**	**August**	**20**
2022	March	1
2022	April	37
2022	May	141
**2022**	**June**	**191**
**2022**	**July**	**83**
**2022**	**August**	**15**

Table S2.2: summary of all 17 climatic, habitat, topographic and anthropogenic variables considered for modelling. Arrows indicate where the same ecological justification, data source, or resolution applies to subsequent variables.

category	variable	description	ecological justification	data source	spatial resolution	temporal resolution
Climatic	Mean spring temperature	Mean maximum temperature of the spring period (March-June)	Spring temperatures affect the phenology, growth and abundance of Lepidoptera (Macphie *et al.*, 2023) and Orthoptera (Buckley *et al.*, 2021; Geppert *et al.*, 2021) – both of which are key prey forcuckoos (Mills *et al.*, 2020).	WorldClim database ver. 4 (Harris *et al.*, 2020)	2.5 arc minutes (~4 km x 4 km)	Yearly
	Total spring precipitation	Total precipitation of the spring period (March-June)	Precipitation in spring influences leaf phenology and in turn, caterpillar growth and abundance (Schöll *et al.*, 2016). Whilst drought can delay and inhibit plant and caterpillar development, excessive rainfall can cause egg and larval drowning, increase disease risk, and reduce caterpillar abundance (Severns *et al.*, 2006; WallisDeVries and van Swaay, 2006; Dennis and Sparks, 2007; Schöll *et al.*, 2016)	↓	↓	↓
	Cumulative winter precipitation	Mean total precipitation of the previous three winters (Dec-Jan)	Winter precipitation of previous years can influence caterpillar abundance (the primary food source of European cuckoos) in the current year (Pollard, 1988; Roy *et al.*, 2001; WallisDeVris *et al.*, 2011)	↓	↓	↓
	Cumulative winter temperature	Mean max temperature of the previous three winters (Dec-Jan)	Extreme winter warmth can have detrimental effects on butterflies at all life stages (McDermott Long *et al.*, 2017) and successive years of such conditions can reduce population sizes (WallisDeVris *et al.*, 2011).	↓	↓	↓
	De Martonne Aridity index (DMAI)	Ratio of total annual precipitation to mean annual temperature plus 10, providing an index of climatic dryness.	Stopover use, particularly in the Mediterranean, is (and will increasingly be) influenced by aridity (Kassara *et al.*, 2025)	↓	↓	↓
	Standardised Precipitation-Evapotranspiration Index (SPEI):<br>SPEI-3 months<br>SPEI-12 months<br>SPEI-36 months	Standardised water balance (precipitation minus potential evapotranspiration) over the previous 3, 12 and 36 months, providing an index of short-, intermediate- and long-term drought relative to the reference period (1901-present).	Contemporaneous drought can reduce prey availability in the current season and may incur direct physiological stress on cuckoos. Equally, the suitability of stopover sites may be reduced if long term drought across previous years have decimated prey populations (Palmer *et al.*, 2017; Marquis *et al.*, 2019; Shan *et al.*, 2024)	Hydro-JULES MSEWP-GLEAM standardised Precipitation Evapotranspiration Index (SPEI) (Gebrechorkos *et al.*, 2023)	~3 arc minutes (~5 km x 5 km)	Monthly
Habitat	Wetland index	Proportional coverage of permanent and temporary wetlands and water bodies.	Cuckoos are able to exploit a range of habitats during the breeding season (Wyllie, 1981; Nakamura and Miyazawa, 1997; Erritzøe *et al.*, 2012). To better understand use of these habitats on migration, *wetland, woodland, grassland, cropland* and *shrubland* indices were included.	Water and Wetness status (CLMS, 2018)	~5 arc minutes (~10 km x10 km)	Study period
	Woodland index	Proportional tree cover.	↓	Copernicus Land Monitoring Scheme (CLMS, 2019)	↓	↓
	Grassland index	Proportional grassland coverage.	↓	↓	↓	↓
	Cropland index	Proportional cropland coverage.	↓	↓	↓	↓
	Shrubland index	Proportional shrubland coverage.	↓	↓	↓	↓
	Habitat heterogeneity	Shannon's diversity index for the five distinct habitat indices outlined above; values normalised (0-1).	Cuckoos often prefer Intermediate forest cover within a mosaic of open patches (de Juana and Garcia, 2015; Williams *et al.*, 2016; Denerley *et al.*, 2019), potentially providing greater foraging opportunities (Moskát *et al.*, 2019).	↓	↓	↓

Topographic	Elevation	Meters above sea level.	Migrating cuckoos are exposed to a wide elevational range (Cheng *et al*., 2025) and as a result of altitudinal variation in habitat and climate, may show elevational preferences at stopover sites.	EarthEnv (Amatulli *et al*., 2018)	~5 arc minutes (~10 km x 10 km)	Study period
Anthropogenic	Hunting risk	Index of hunting pressure on small birds (relative to a feral pigeon), based on insight from 137 local experts across 98 countries.	The hunting of migratory birds is still prevalent across Europe, especially in mediterranean regions, potentially posing a threat to cuckoos (Brochet *et al*., 2016, 2017; Hirschfeld *et al*., 2019).	Relative hunting threat (small birds) (Buchan *et al*., 2022)	5 arc minutes (~10 km x 10 km)	Study period
	Pesticide use	Total cropland pesticide use for each European country, multiplied by the cropland index (0-1)	Intensive use of pesticides across Europe has reduced abundance of invertebrates and in turn, insectivorous birds (Rigal *et al*., 2023). Indeed, lowland agricultural areas of the UK - where agricultural intensification has been greatest - have experienced the most severe cuckoo declines (Denerley et al., 2019). Thus, suitability of stopover sites may be associated with pesticide application.	FAO (2025) and Copernicus land monitoring scheme (CLMS, 2019)	5 arc minutes (~10 km x 10 km)	Yearly

Table S2.3: Variable Inflation Factor (VIF) values for all variables retained after correlation analysis. VIF values indicate the degree to which the variance of each variable is inflated by correlation with other variables. Values of 1 indicate no collinearity; values <5 indicate low-to-moderate correlation (Daoud, 2017).

**Variable**	**VIF value**
spring_temp	3.507978
SPEI_36	1.423738
SPEI_12	1.951907
SPEI_03	1.407244
aridity	2.585356
wetland_index	1.376589
treecover_index	4.427713
grassland_index	2.905756
shrubland_index	3.123336
cropland_index	4.71415
habitat heterogeneity	1.823822
hunting_risk	2.503619
elevation	2.72953

Table S2.4: Variable Inflation Factor (VIF) values for all variables retained for sensitivity analysis. VIF values indicate the degree to which the variance of each variable is inflated by correlation with other variables. Values of 1 indicate no collinearity; values <5 indicate low-to-moderate correlation (Daoud, 2017).

**Variable**	**VIF value**
winter_temp	3.743614
winter_precip	1.886296
spring_precip	2.037124
SPEI_36	1.532971
SPEI_12	2.045747
SPEI_03	1.483821
wetland_index	1.330291
treecover_index	3.48596
grassland_index	2.805449
shrubland_index	3.372519
pesticide_use	3.089363
habitat heterogeneity	1.715598
hunting_risk	2.821163
elevation	3.161947

Table S2.5: mean Area Under the Receiver Operator Curve (AUC) values for SE MaxEnt models tunned with different combinations of feature class ('l' = linear, 'lq' = linear quadratic, 'lqh' = linear quadratic hinge, 'lqph' = linear quadratic product hinge) and regularisation multiplier (0.5-3). AUC values are the mean from 5-fold cross validation tunning. Combinations are ordered from highest-to-lowest performing, based on AUC values.

**feature class**	**regularisation multiplier**	**mean AUC**
lqh	0.5	0.81
lqh	1	0.81
lqph	0.5	0.81
lqph	1	0.80
lqh	2	0.80
lqph	2	0.79
lqph	3	0.79
lqh	3	0.79
lq	0.5	0.78
lq	1	0.78
lq	2	0.77
lq	3	0.76
l	2	0.75
l	3	0.75
l	1	0.75
l	0.5	0.75

Table S2.6: mean Area Under the Receiver Operator Curve (AUC) values for SW MaxEnt models tunned with different combinations of feature class ('l' = linear, 'lq' = linear quadratic, 'lqh' = linear quadratic hinge, 'lqph' = linear quadratic product hinge) and regularisation multiplier (0.5-3). AUC values are the mean from 5-fold cross validation tunning. Combinations are ordered from highest-to-lowest performing, based on AUC values.

feature class	regularisation multiplier	mean AUC
lqph	0.5	0.82
lqph	1	0.81
lqh	0.5	0.80
lqh	1	0.78
lqph	2	0.78
lqh	2	0.78
lqph	3	0.77
lqh	3	0.76
lq	0.5	0.74
lq	1	0.74
lq	2	0.73
lq	3	0.72
l	0.5	0.70
l	1	0.70
l	2	0.70
l	3	0.69

Table S2.7: Area Under The Receiver Operating Characteristic Curve (AUC) and True Skill Statistic (TSS) values for all models, including MaxEnt. Values are the mean from 10-fold cross validation, with 95% confidence intervals in parentheses. Dashed lines separate models by migration route and the inclusion of latitude ('+lat'); for each group of models, the highest AUC and TSS values are in bold.

migration route	model	mean AUC	mean TSS
east	BRT	0.83 (0.80-0.85)	0.57 (0.52-0.62)
east	RF	**0.85 (0.83-0.88)**	**0.61 (0.56-0.66)**
east	MaxEnt	0.80 (0.76-0.83)	0.51 (0.44-0.58)
east	ensemble	0.84 (0.81-0.87)	0.58 (0.53-0.63)
east	BRT + lat	0.85 (0.82-0.87)	0.58 (0.54-0.63)
east	RF + lat	**0.87 (0.84-0.89)**	**0.65 (0.61-0.69)**
east	MaxEnt	0.80 (0.76-0.83)	0.50 (0.43-0.56)
east	ensemble + lat	0.85 (0.82-0.88)	0.59 (0.54-0.64)
west	BRT	0.79 (0.78-0.80)	0.50 (0.47-0.54)
west	RF	**0.85 (0.83-0.88)**	**0.64 (0.59-0.69)**
west	MaxEnt	0.76 (0.74-0.78)	0.47 (0.44-0.50)
west	ensemble	0.82 (0.81-0.84)	0.56 (0.53-0.59)
west	BRT + lat	0.82 (0.80-0.84)	0.57 (0.53-0.61)
west	RF + lat	**0.87 (0.86-0.89)**	**0.66 (0.63-0.70)**
west	MaxEnt	0.79 (0.76-0.82)	0.51 (0.46-0.56)
west	ensemble + lat	0.85 (0.83-0.87)	0.61 (0.55-0.66)

Table S2.8: Area Under The Receiver Operating Characteristic Curve (AUC), True Skill Statistic (TSS), and maximum training sensitivity plus specificity (MTSS) threshold for all models in the variable sensitivity analysis. Values are the mean from 10-fold cross validation, with 95% confidence intervals in parentheses. Dashed lines separate models by migration route and the inclusion of latitude ('+lat'); for each group of models, the highest AUC and TSS values are in bold.

migration route	model	mean AUC	mean TSS	mean MTSS threshold
east	BRT	0.85 (0.83-0.88)	0.62 (0.56-0.67)	0.46 (0.39-0.54)
east	RF	**0.86 (0.83-0.89)**	**0.66 (0.59-0.72)**	0.22 (0.19-0.25)
east	ensemble	**0.86 (0.84-0.89)**	0.64 (0.58-0.70)	0.39 (0.33-0.45)
east	BRT + lat	0.86 (0.83-0.89)	0.62 (0.56-0.67)	0.48 (0.42-0.55)
east	RF + lat	**0.87 (0.84-0.90)**	**0.67 (0.61-0.74)**	0.24 (0.20-0.27)
east	ensemble + lat	**0.87 (0.84-0.88)**	**0.67 (0.60-0.74)**	0.36 (0.30-0.42)
west	BRT	0.80 (0.77-0.84)	0.53 (0.48-0.58)	0.40 (0.33-0.47)
west	RF	**0.87 (0.84-0.89)**	**0.66 (0.59-0.72)**	0.23 (0.19-0.26)
west	ensemble	0.85 (0.82-0.88)	0.62 (0.58-0.67)	0.33 (0.30-0.37)
west	BRT + lat	0.84 (0.80-0.87)	0.59 (0.53-0.64)	0.36 (0.33-0.40)
west	RF + lat	**0.89 (0.87-0.91)**	**0.69 (0.65-0.74)**	0.23 (0.19-0.26)
west	ensemble + lat	0.87 (0.85-0.90)	0.66 (0.61-0.71)	0.34 (0.30-0.38)
