# Supplementary material for: Drivers of Stopover Distribution Across Two Autumn Migration Routes of a Long‐Distance Afro‐Palearctic Migrant, the Common Cuckoo ( Cuculus canorus )
Source: Ecol Evol. 2026 Aug 4;16(8):e74083. doi: 10.1002/ece3.74083 (PMC13435883; doi:10.1002/ece3.74083)
Supplement: Supplementary file 2 [file ECE3-16-e74083-s002.pdf]

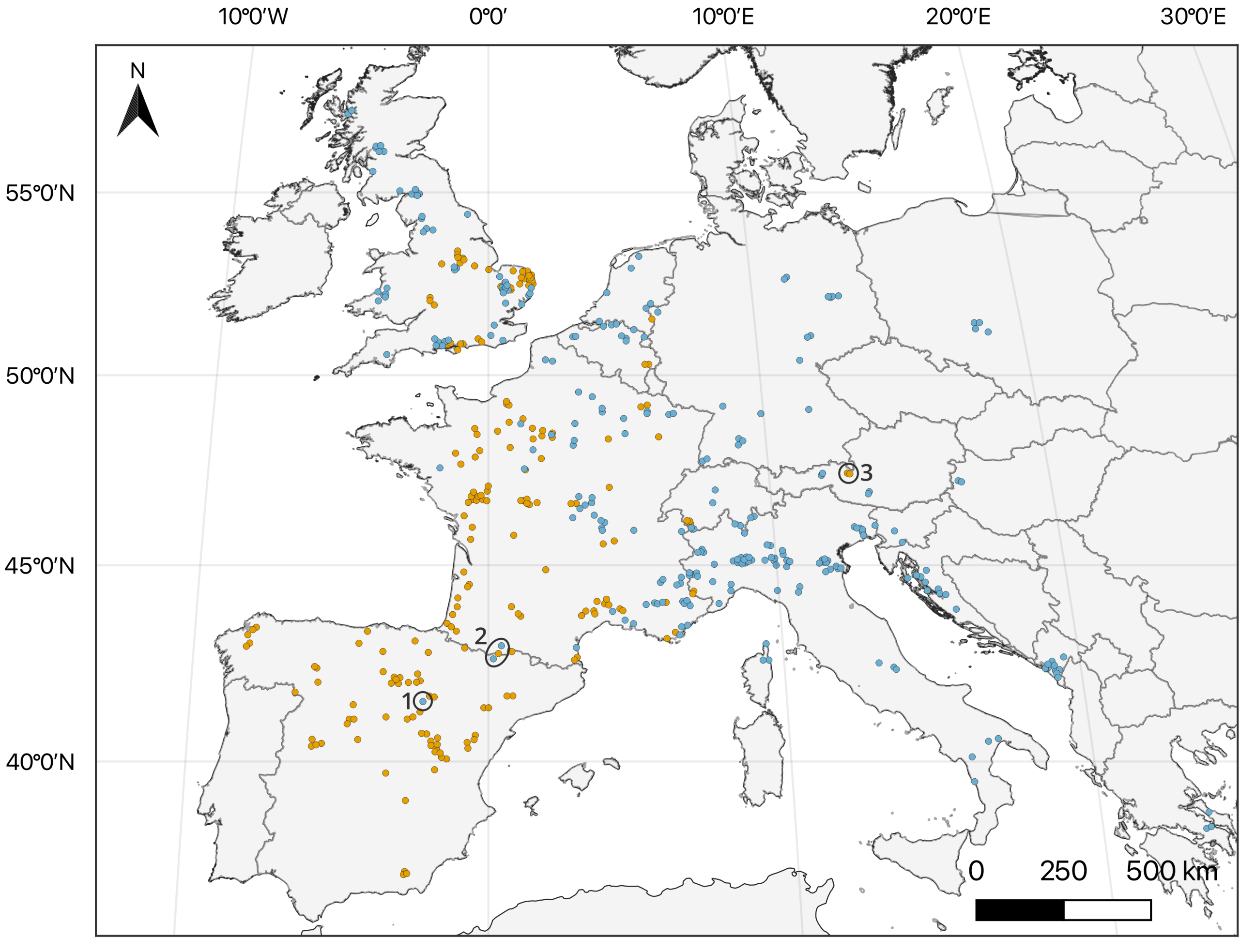


Figure S3.1: atypical stopovers made by two south easternly migrating birds (circles 1 and 2) and one south westerly migrating individual (circle 3). Atypicals were manually removed prior to analysis. The plot is projected using the Mollweide projection (ESRI: 54009).

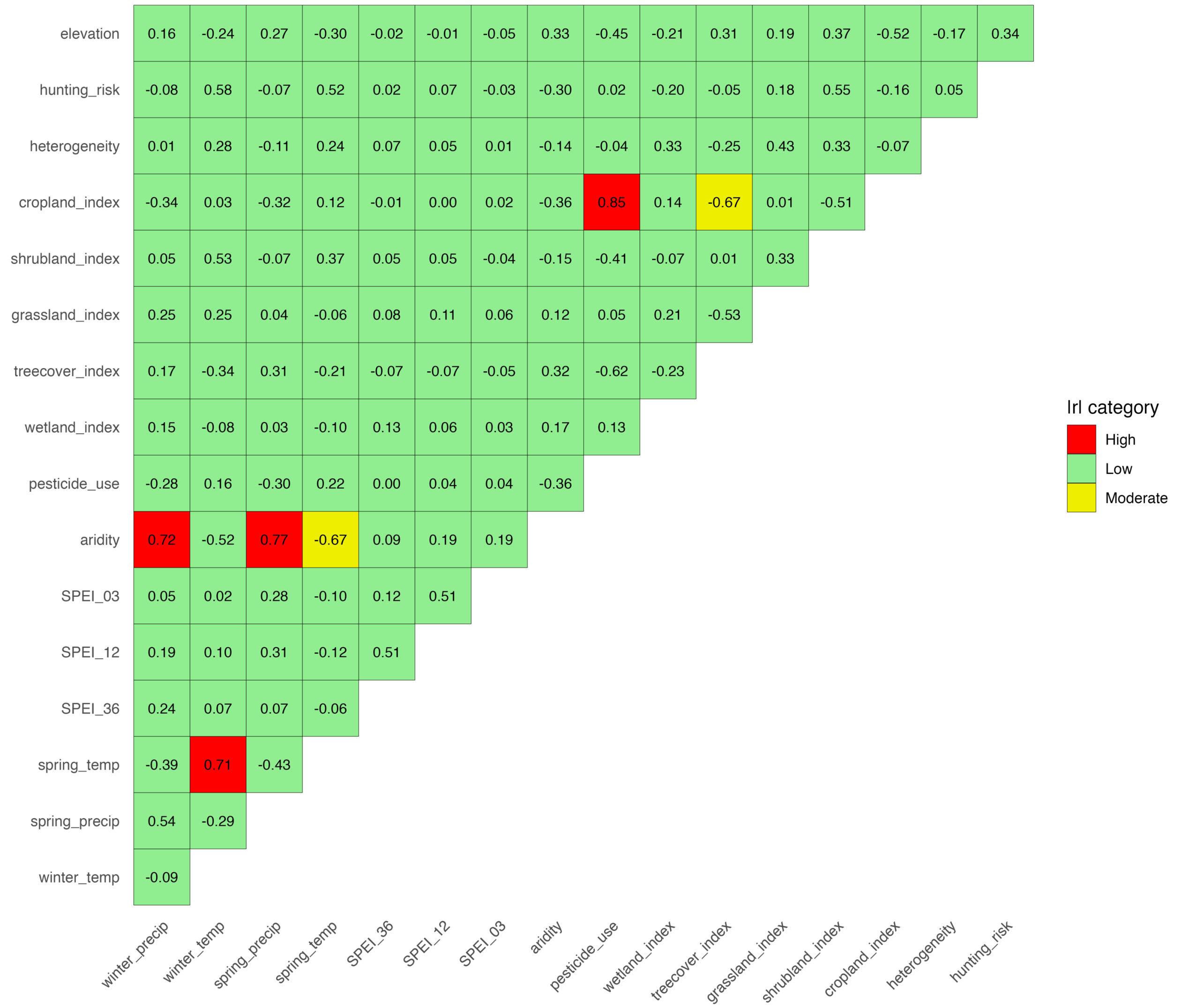


Figure S3.2: correlation matrix for all environmental variables considered for modelling. |*r*| values are categorised into **high** (|*r*| ≥0.7), **moderate** (0.7> |*r*| ≥ 0.6) or **low** (|*r*| < 0.6).

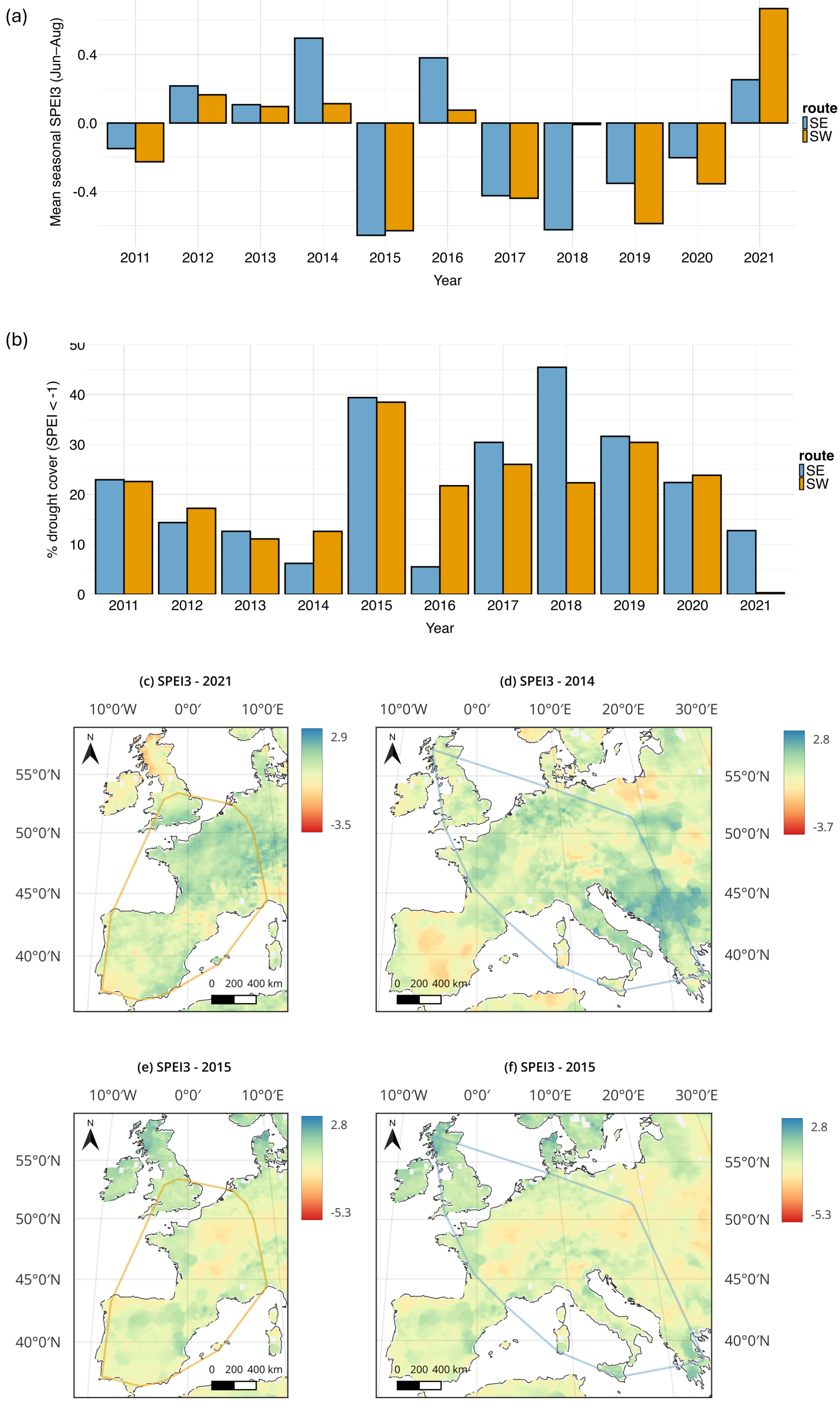


Figure S3.3: (a) mean SPEI3 values, and (b) percentage drought cover (SPEI3 values < -1) along eastern and western migration routes for each year in the study period. Heat maps show the SPEI3 drought values across the eastern and western routes for years of low (c & d) and high (e & f) SPEI3 drought. Polygons denote the areas in which models were trained. SPEI maps are projected using the Mollweide projection (ESRI: 54009).

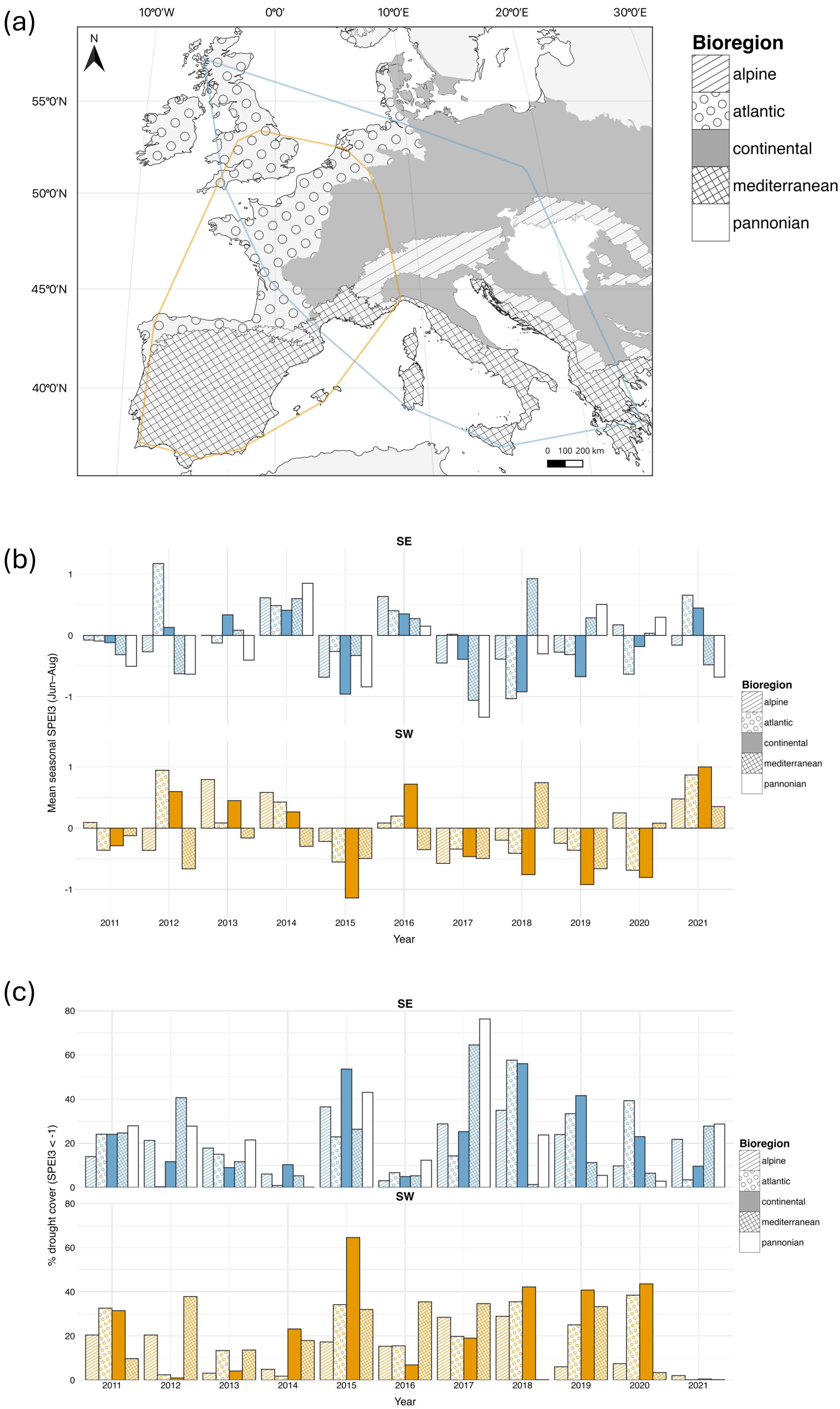


Figure S3.4: (a) bioregions within the eastern and western study areas. (b) mean SPEI3 values, and (c) percentage drought cover (SPEI3 values < -1) for each bioregion. The bioregion plot is projected using the Mollweide projection (ESRI: 54009).

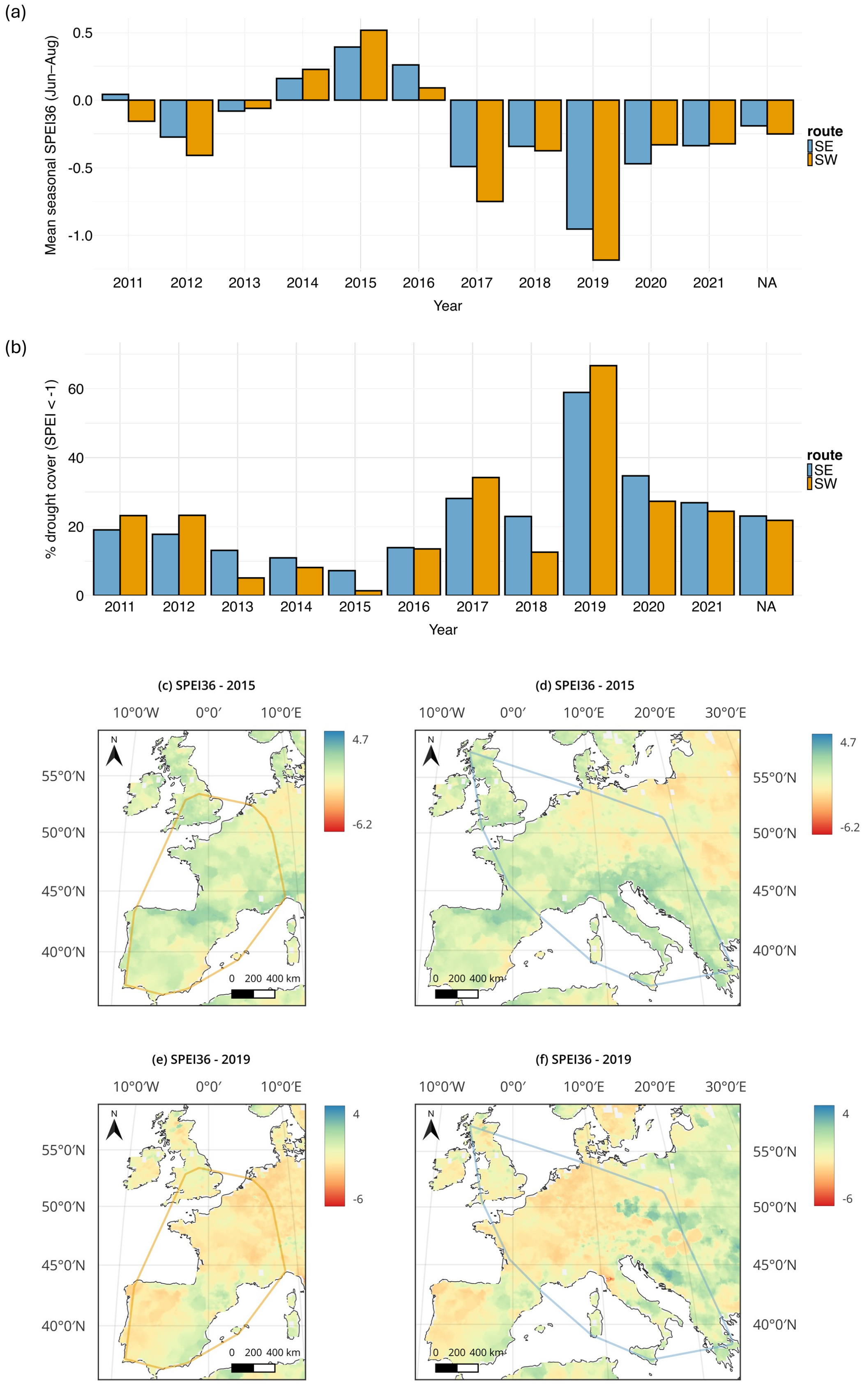


Figure S3.5: (a) mean SPEI36 values, and (b) percentage drought cover (SPEI36 values < -1) along eastern and western migration routes for each year in the study period. Heat maps show the SPEI36 drought values across the eastern and western routes for years of low (c & d) and high (e & f) SPEI36 drought. Polygons denote the areas in which models were trained. SPEI plots are projected using the Mollweide projection (ESRI: 54009).

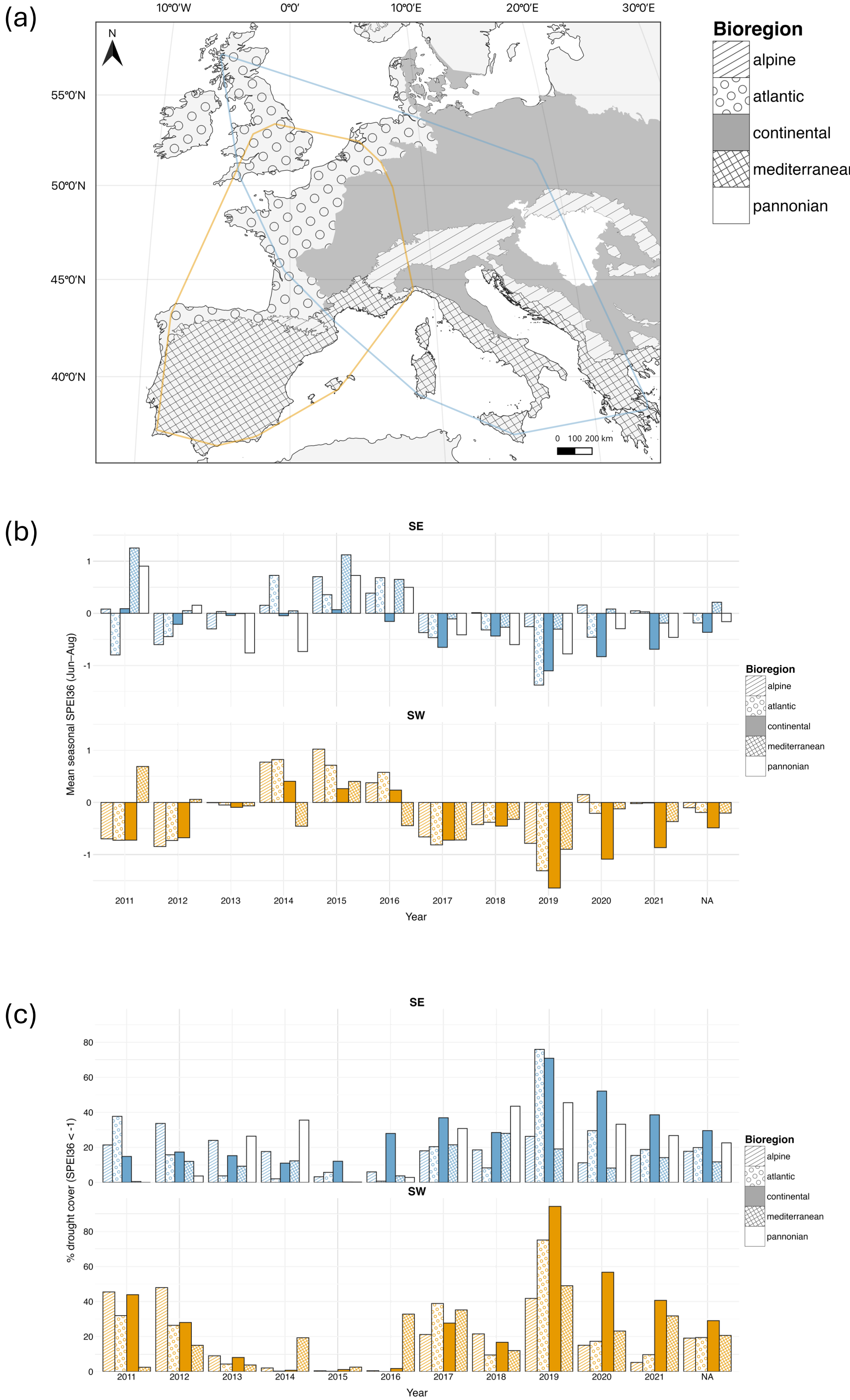


Figure S3.6: (a) bioregions within the eastern and western study areas. (b) mean SPEI36 values, and (c) percentage drought cover (SPEI36 values < -1) for each bioregion. The bioregion plot is projected using the Mollweide projection (ESRI: 54009).

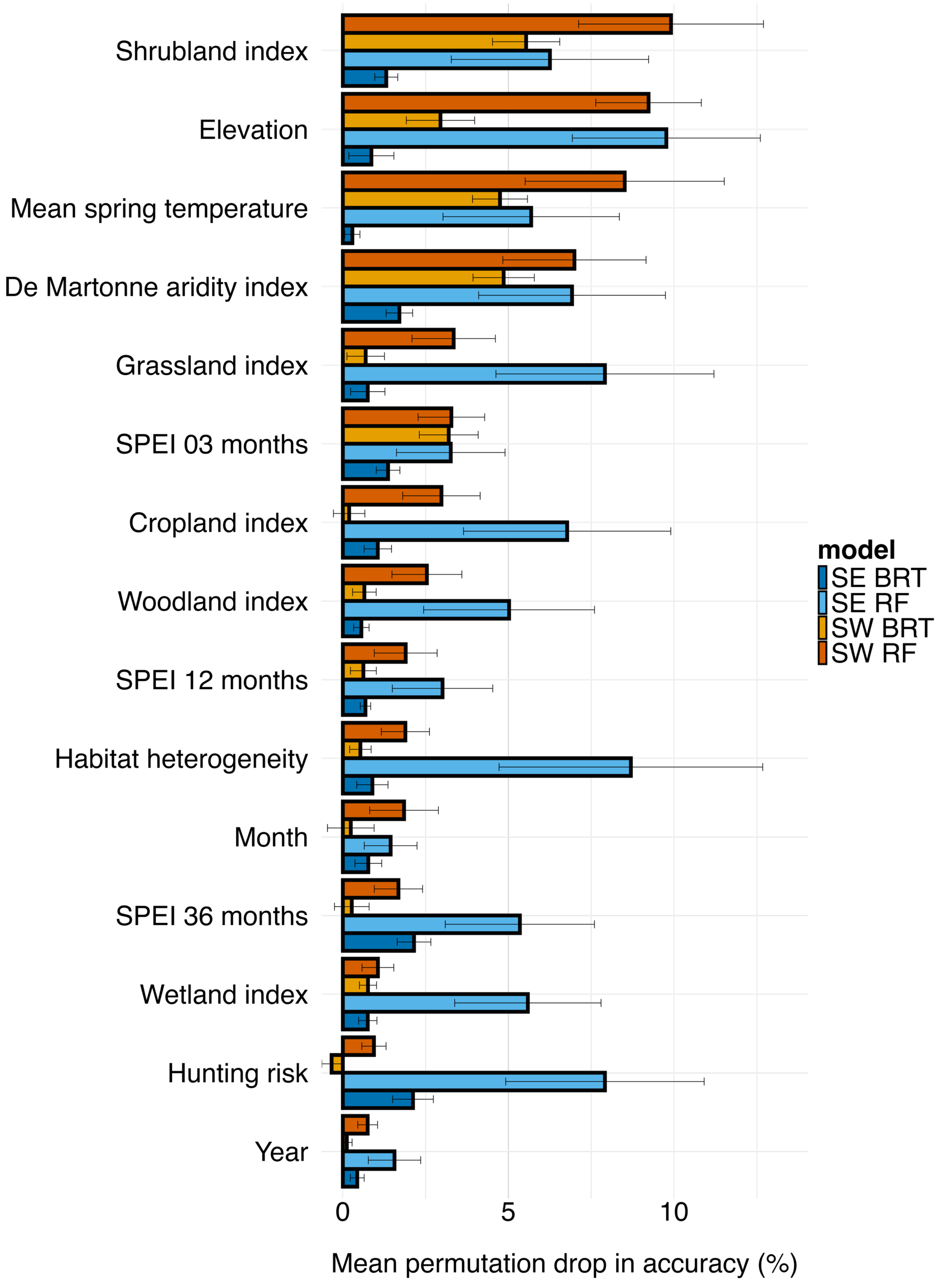


Figure S3.7: permutation importance of variables for constituent RF and BRT models. Values represent the mean percentage drop in accuracy following permutation across 10-fold cross validation, with whiskers indicating upper and lower 95% confidence intervals. Bar colour denotes the model and route (see key). Variables are ranked in descending order for the western route, with their comparative importance for the eastern route.

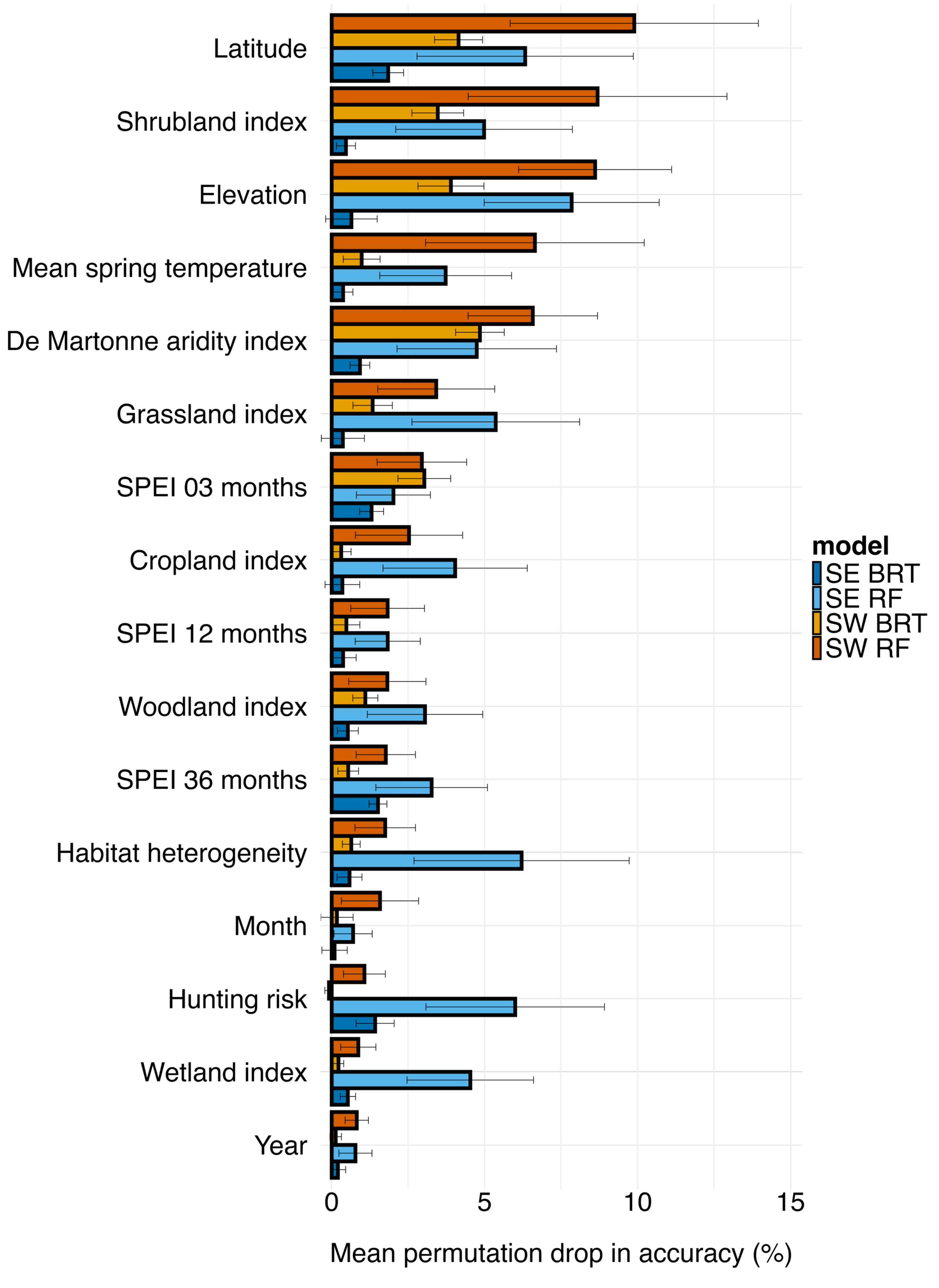


Figure S3.8: permutation importance of variables for constituent RF and BRT models, with latitude included as a predictor. Values represent the mean percentage drop in accuracy following permutation across 10-fold cross validation, with whiskers indicating upper and lower 95% confidence intervals. Bar colour denotes the model and route (see key). Variables are ranked in descending order for the western route, with their comparative importance for the eastern route.

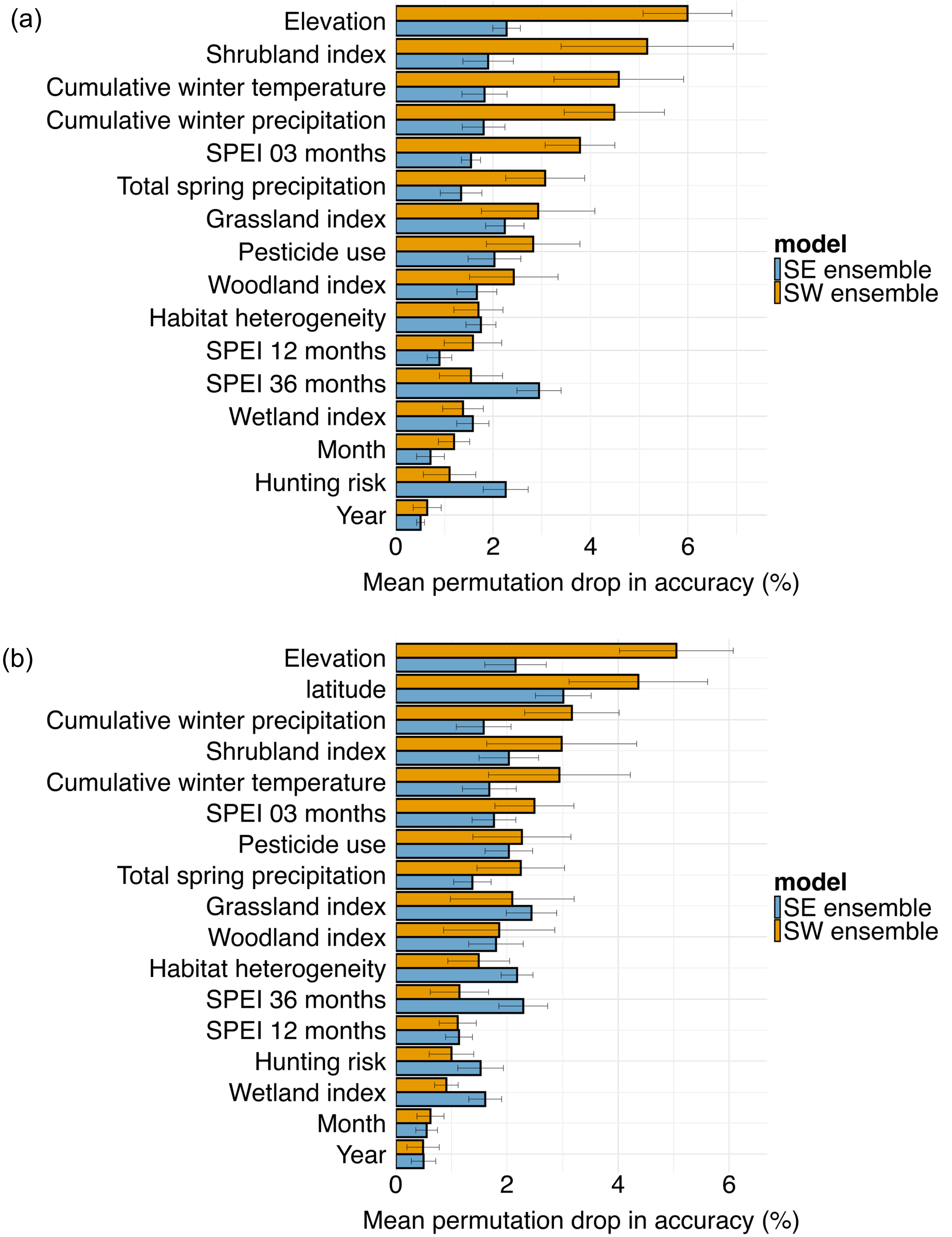


Figure S3.9: percentage drop in accuracy following permutation of each variable for (a) ensemble sensitivity models excluding latitude, and (b) ensemble sensitivity models including latitude as a predictor. Values represent the mean from 10-fold cross validation, with whiskers indicating upper and lower 95% confidence intervals. Variables are ranked in descending order for the **western** route, with their comparative importance for the **eastern** route.

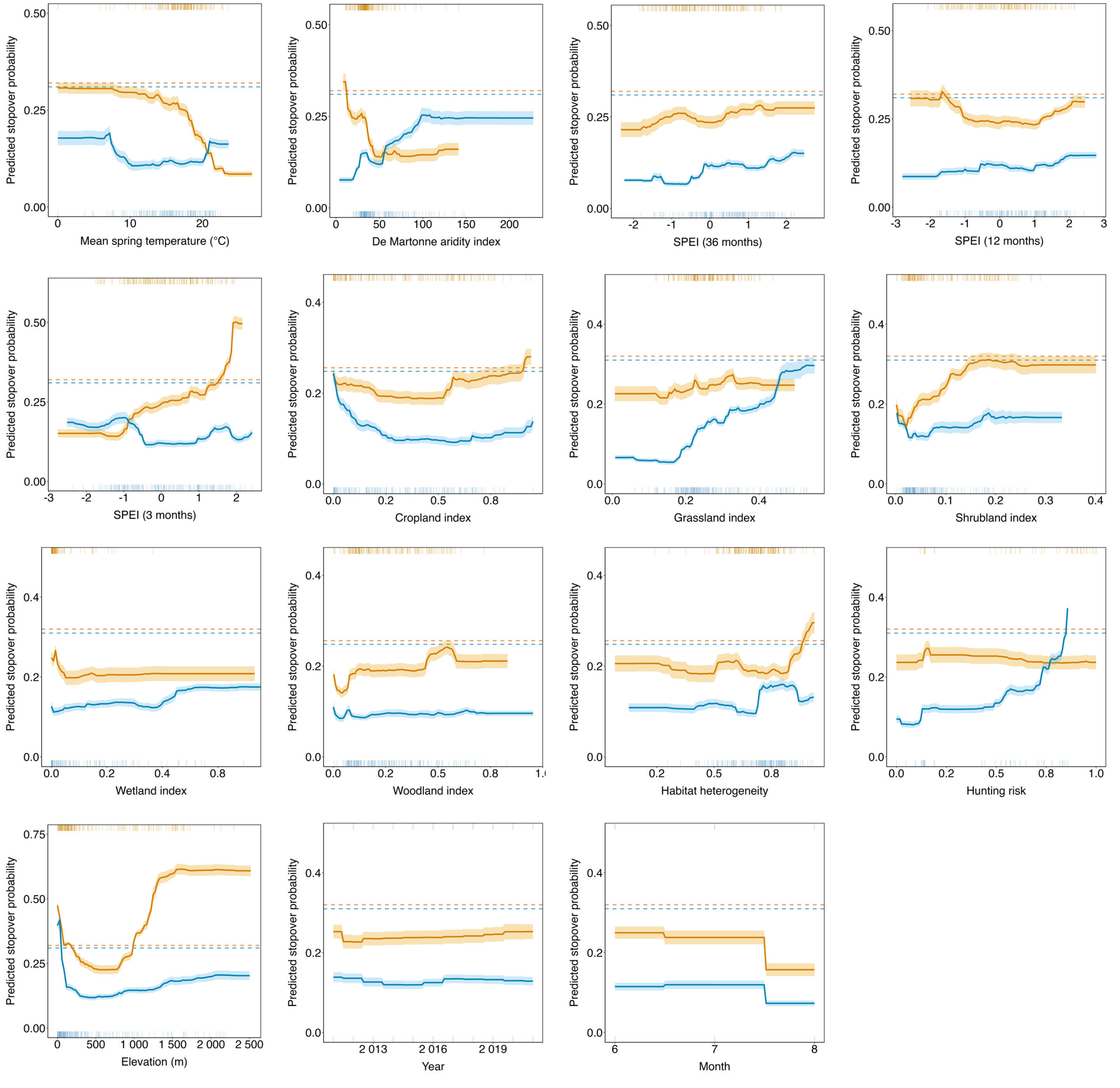


Figure S3.10: response curves (with 95% confidence intervals) for all variables included in the eastern (**blue**) and western (**orange**) ensemble models. **Blue** and **orange** dashed lines indicate the mean max TSS threshold for eastern and western models, respectively. Rugs denote true stopovers.

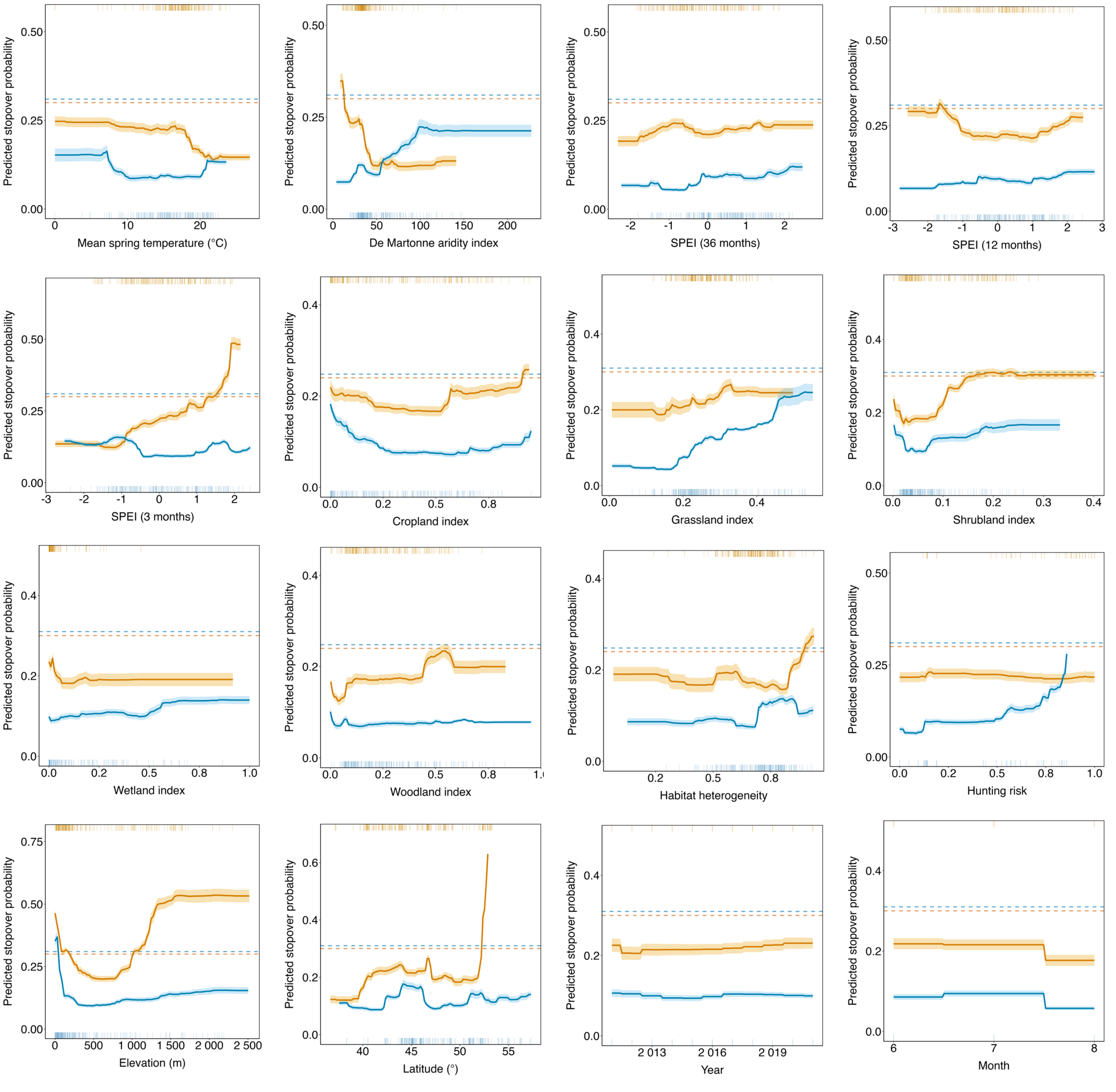


Figure S3.11: response curves (with 95% confidence intervals) for all variables included in the eastern (**blue**) and western (**orange**) ensemble models, with latitude included as a predictor. **Blue** and **orange** dashed lines indicate the mean max TSS threshold for eastern and western models, respectively. Rugs denote true stopovers.

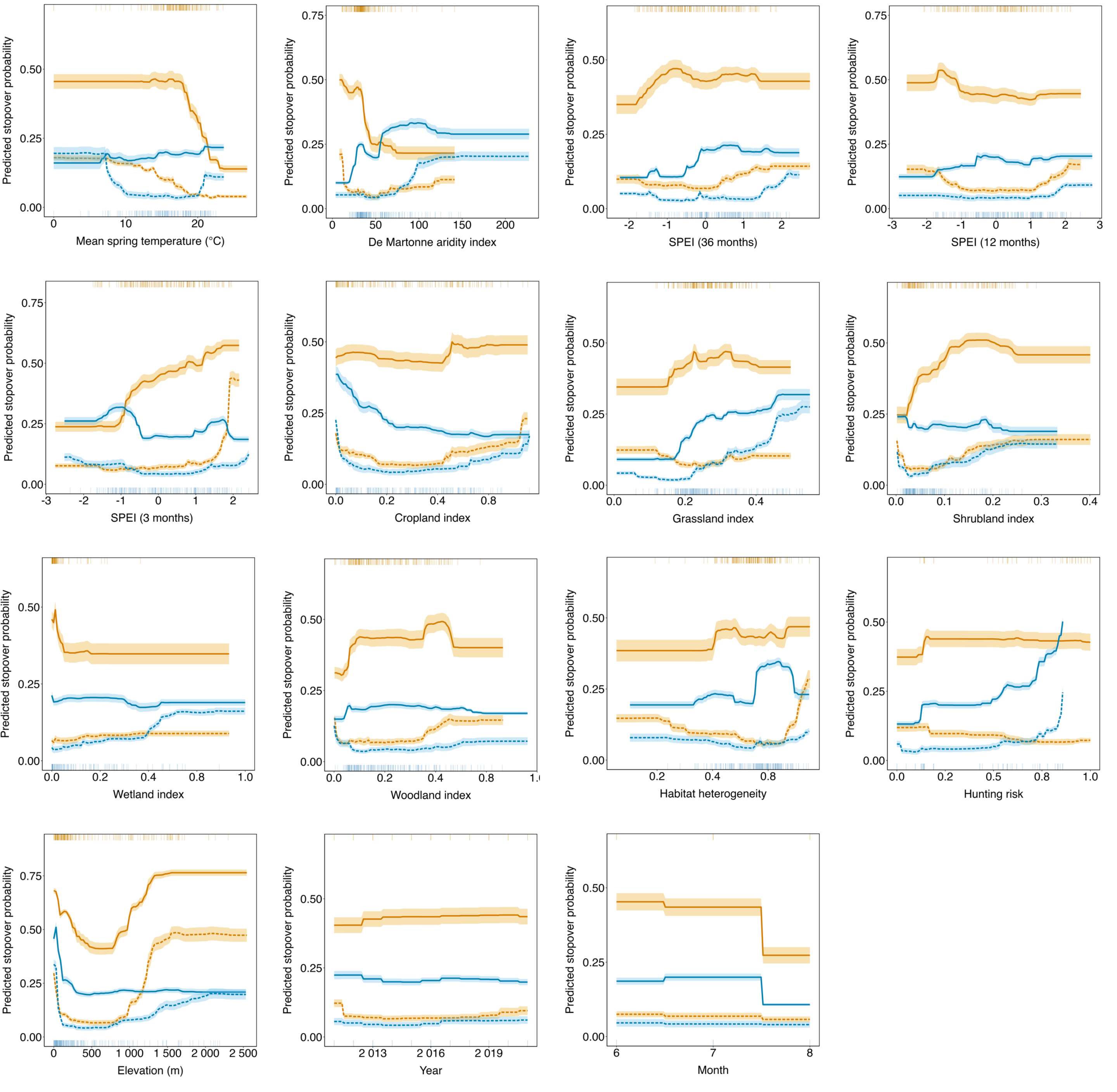


Figure S3.12: response curves (with 95% confidence intervals) for all variables included in the eastern BRT (**solid blue**), eastern RF (**dashed blue**), western BRT (**solid orange**) and western RF (**dashed orange**) models. Rugs denote true stopovers.

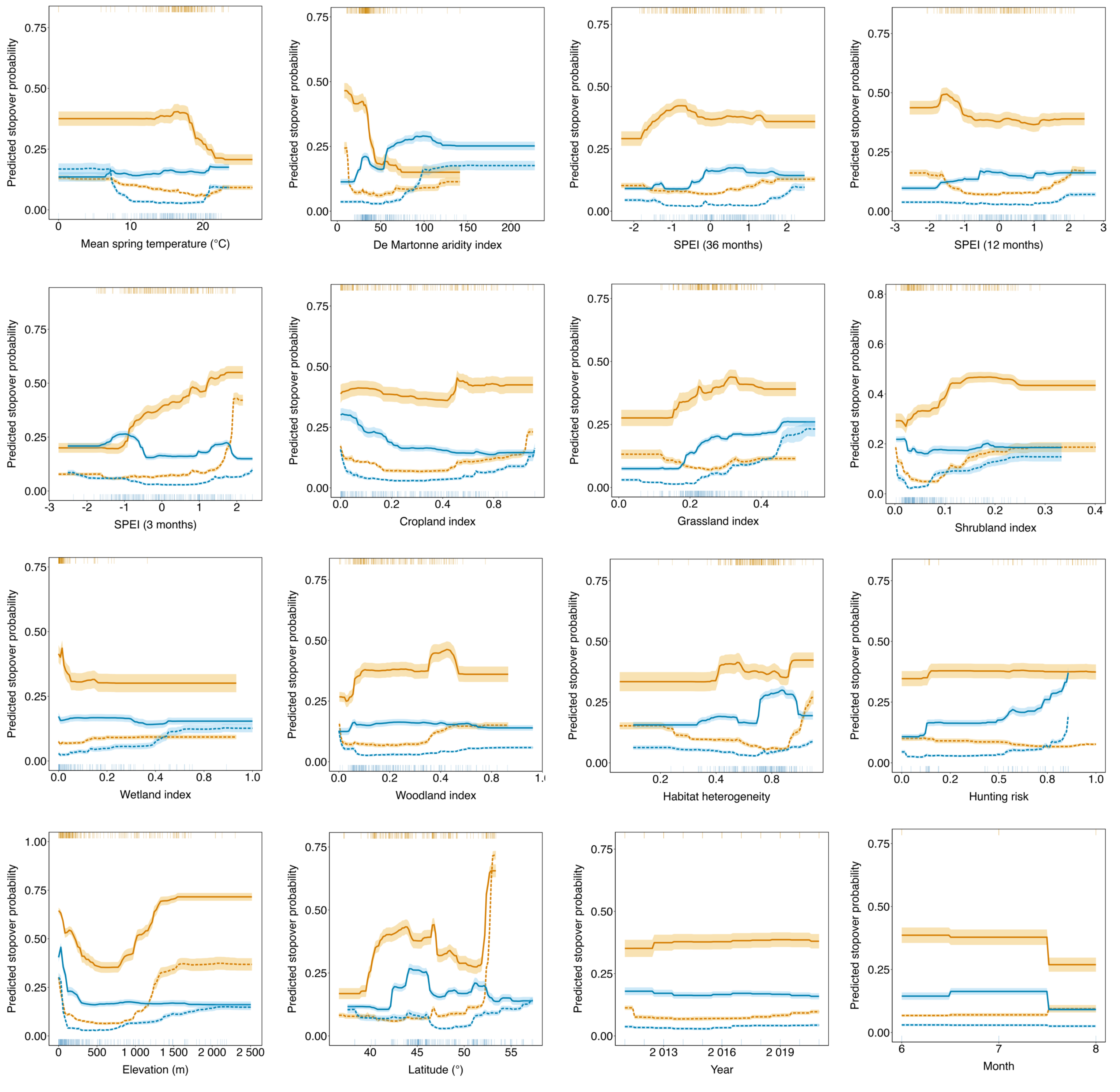


Figure S3.13: response curves (with 95% confidence intervals) for all variables included in the eastern BRT (**solid blue**), eastern RF (**dashed blue**), western BRT (**solid orange**) and western RF (**dashed orange**) models, with latitude included as a predictor. Rugs denote true stopovers.

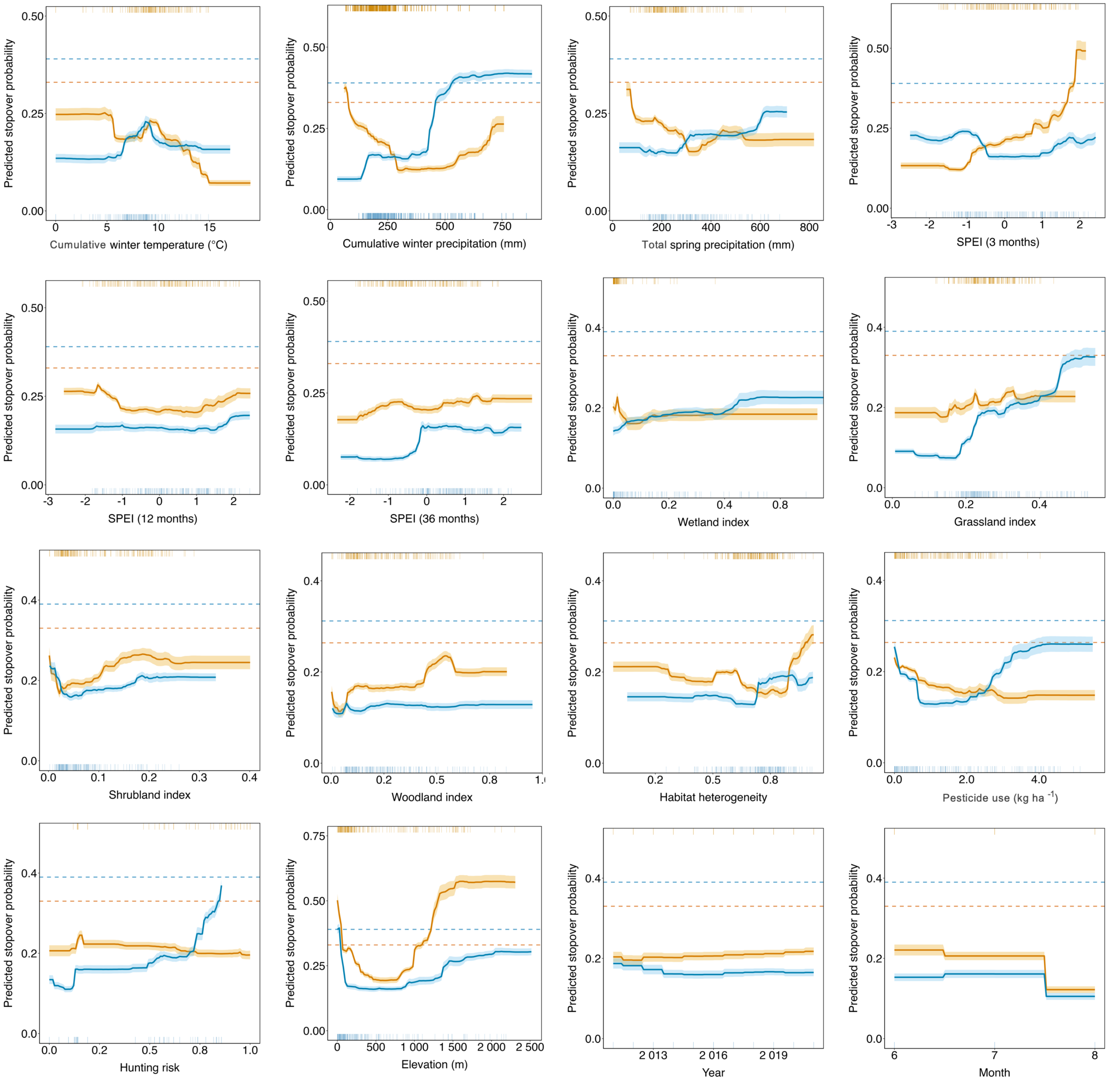


Figure S3.14: response curves (with 95% confidence intervals) for all variables included in the eastern (**blue**) and western (**orange**) ensemble sensitivity models. **Blue** and **orange** dashed lines indicate the mean max TSS threshold for eastern and western models, respectively. Rugs denote true stopovers.

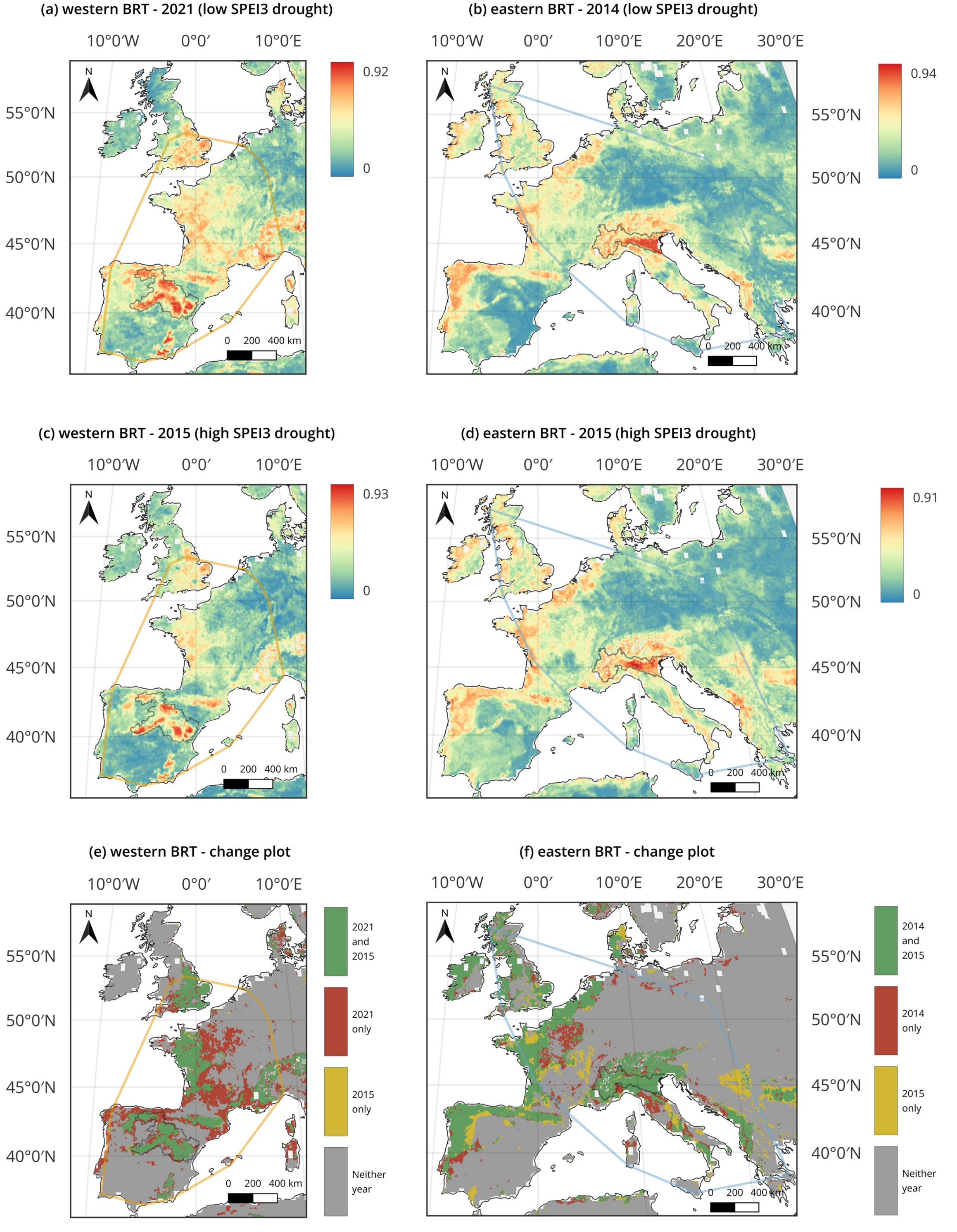


Figure S3.15: Boosted Regression Tree (BRT) spatial predictions for the western (a, c, e) and eastern (b, d, f) migration routes. Heat maps for years of low (a & b) and high (c & d) SPEI3 drought indicate stopover probability (see keys). Key regions of predicted stopover use are outlined by black borders. For change maps (e & f), colours indicate whether stopovers were predicted across one, both or neither years (see keys). Polygons denote the area in which models were trained. Maps are projected using the Mollweide projection (ESRI: 54009).

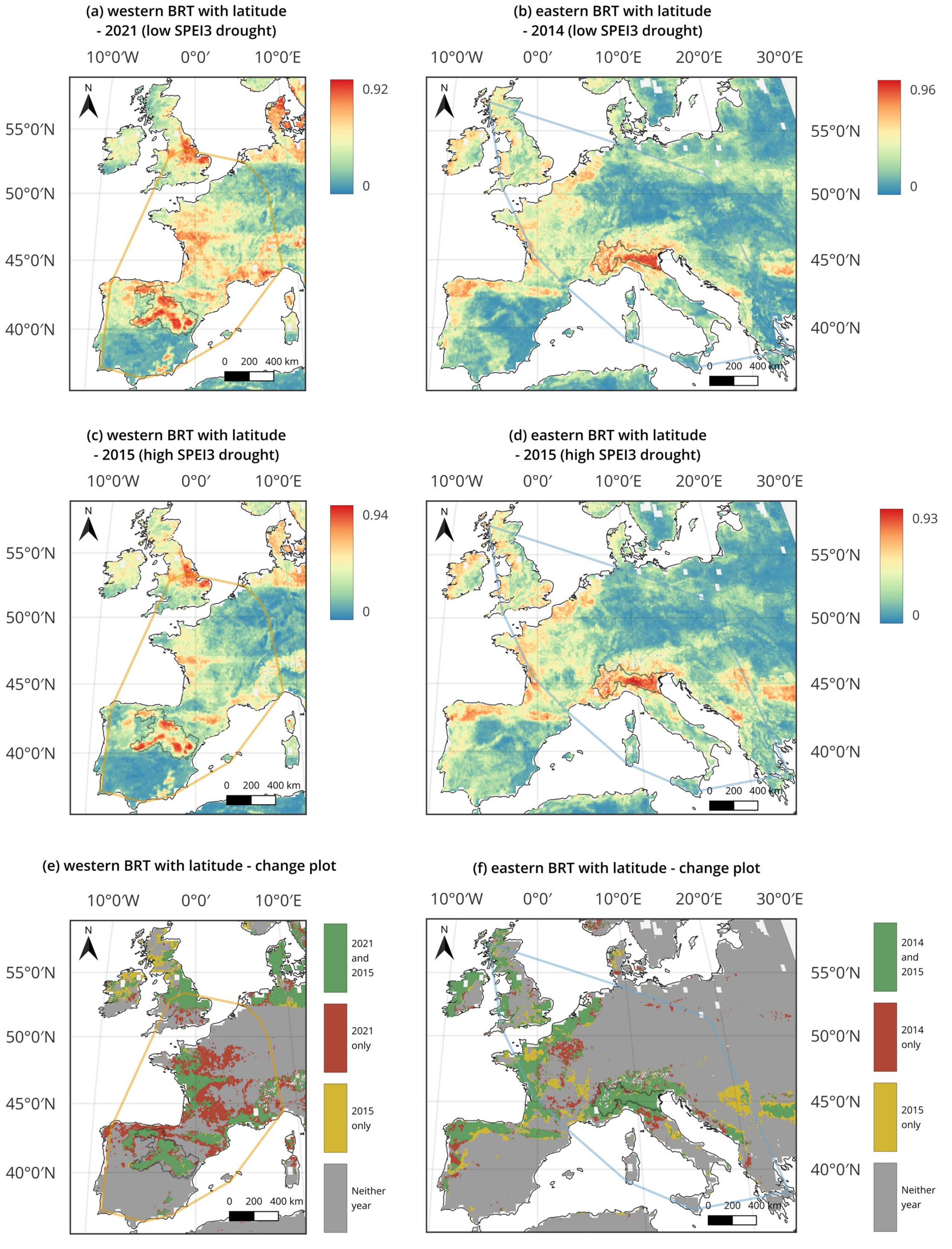


Figure S3.16: Boosted Regression Tree (BRT) spatial predictions for the western (a, c, e) and eastern (b, d, f) migration routes, with latitude included as a predictor. Heat maps for years of low (a & b) and high (c & d) SPEI3 drought indicate stopover probability (see keys). Key regions of predicted stopover use are outlined by black borders. For change maps (e & f), colours indicate whether stopovers were predicted across one, both or neither years (see keys). Polygons denote the area in which models were trained. Maps are projected using the Mollweide projection (ESRI: 54009).

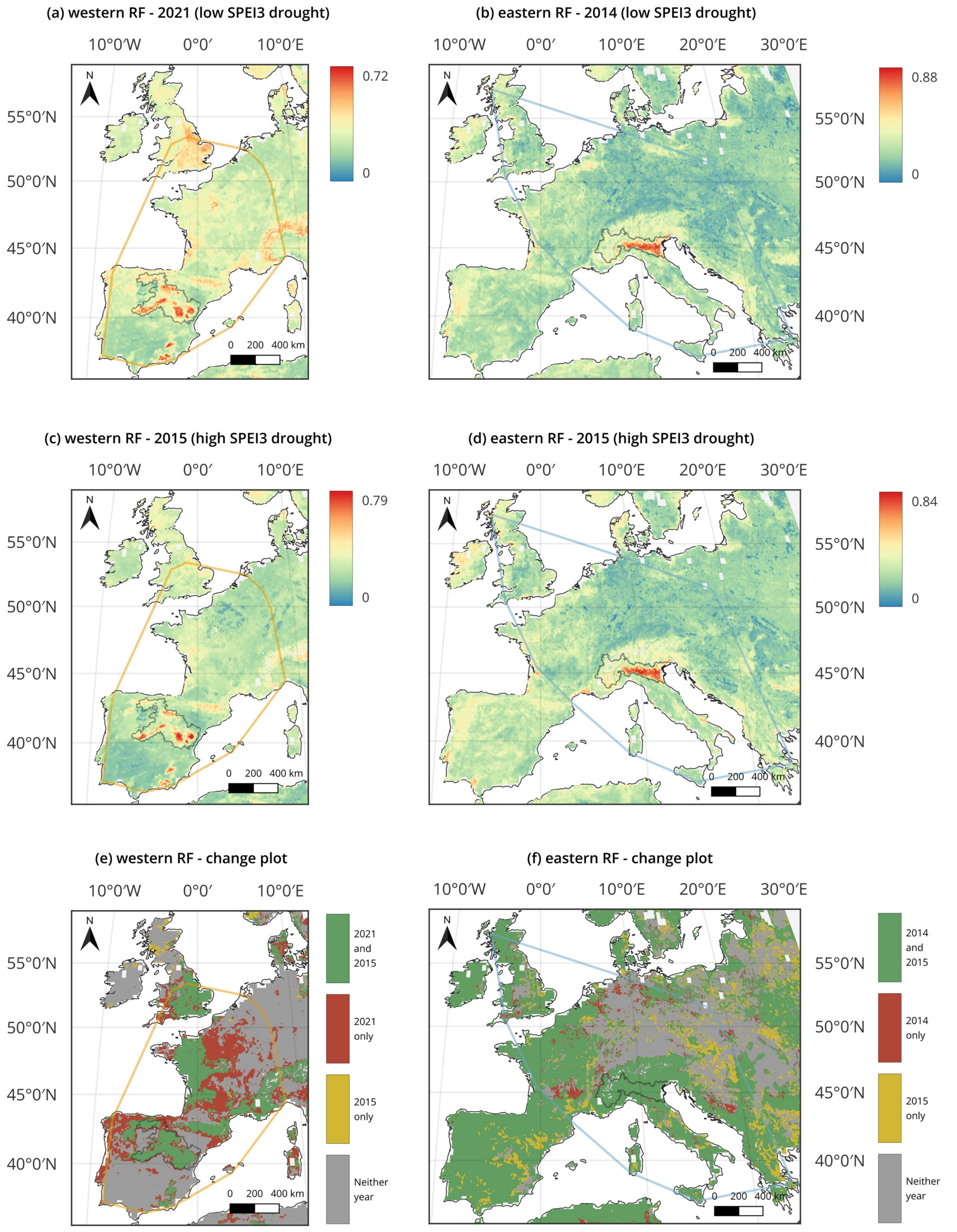


Figure S3.17: Random Forest (RF) spatial predictions for the western (a, c, e) and eastern (b, d, f) migration routes. Heat maps for years of low (a & b) and high (c & d) SPEI3 drought indicate stopover probability (see keys). Key regions of predicted stopover use are outlined by black borders. For change maps (e & f), colours indicate whether stopovers were predicted across one, both or neither years (see keys). Polygons denote the area in which models were trained. Maps are projected using the Mollweide projection (ESRI: 54009).

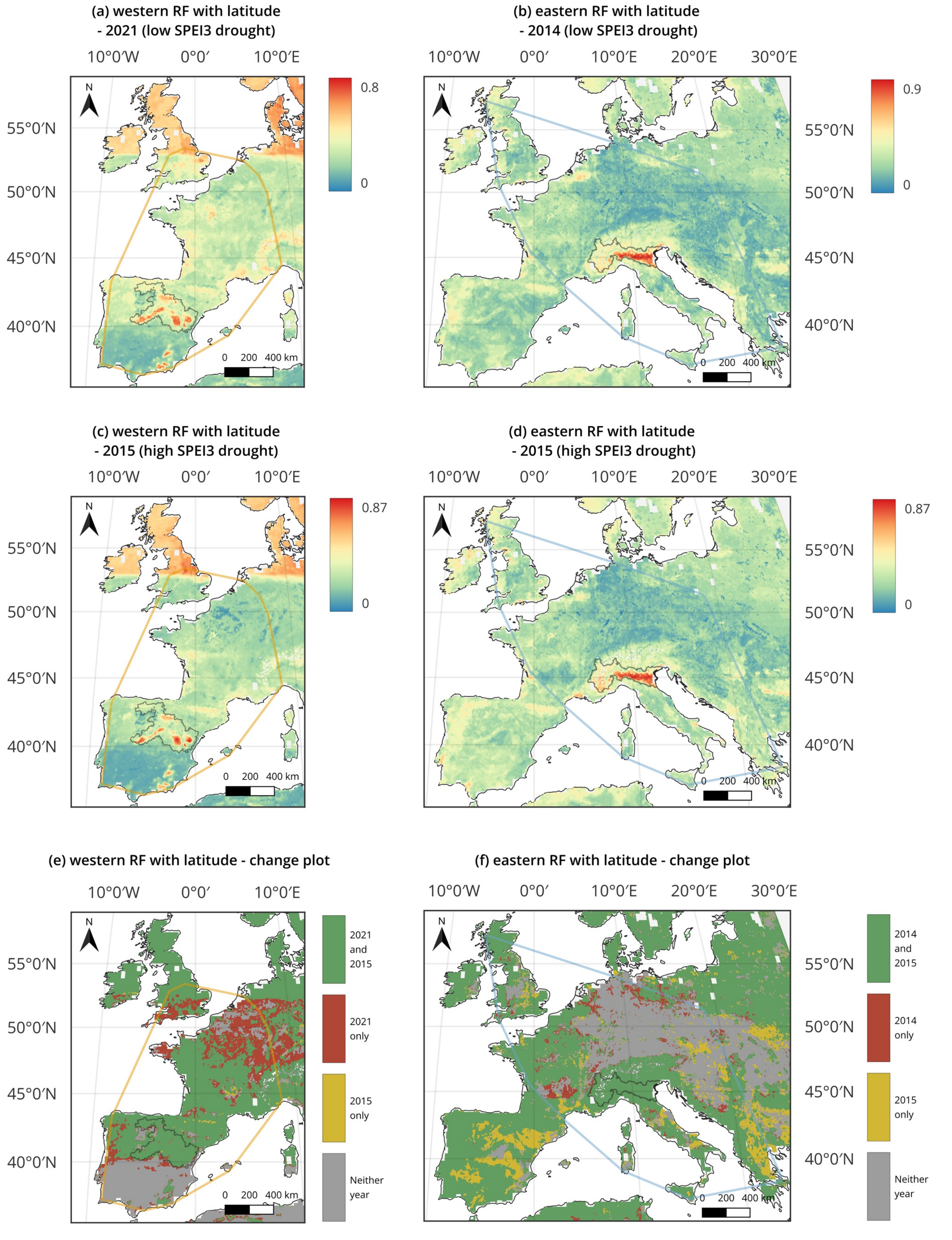


Figure S3.18: Random Forest (RF) spatial predictions for the western (a, c, e) and eastern (b, d, f) migration routes, with latitude included as a predictor. Heat maps for years of low (a & b) and high (c & d) SPEI3 drought indicate stopover probability (see keys). Key regions of predicted stopover use are outlined by black borders. For change maps (e & f), colours indicate whether stopovers were predicted across one, both or neither years (see keys). Polygons denote the area in which models were trained. Maps are projected using the Mollweide projection (ESRI: 54009).

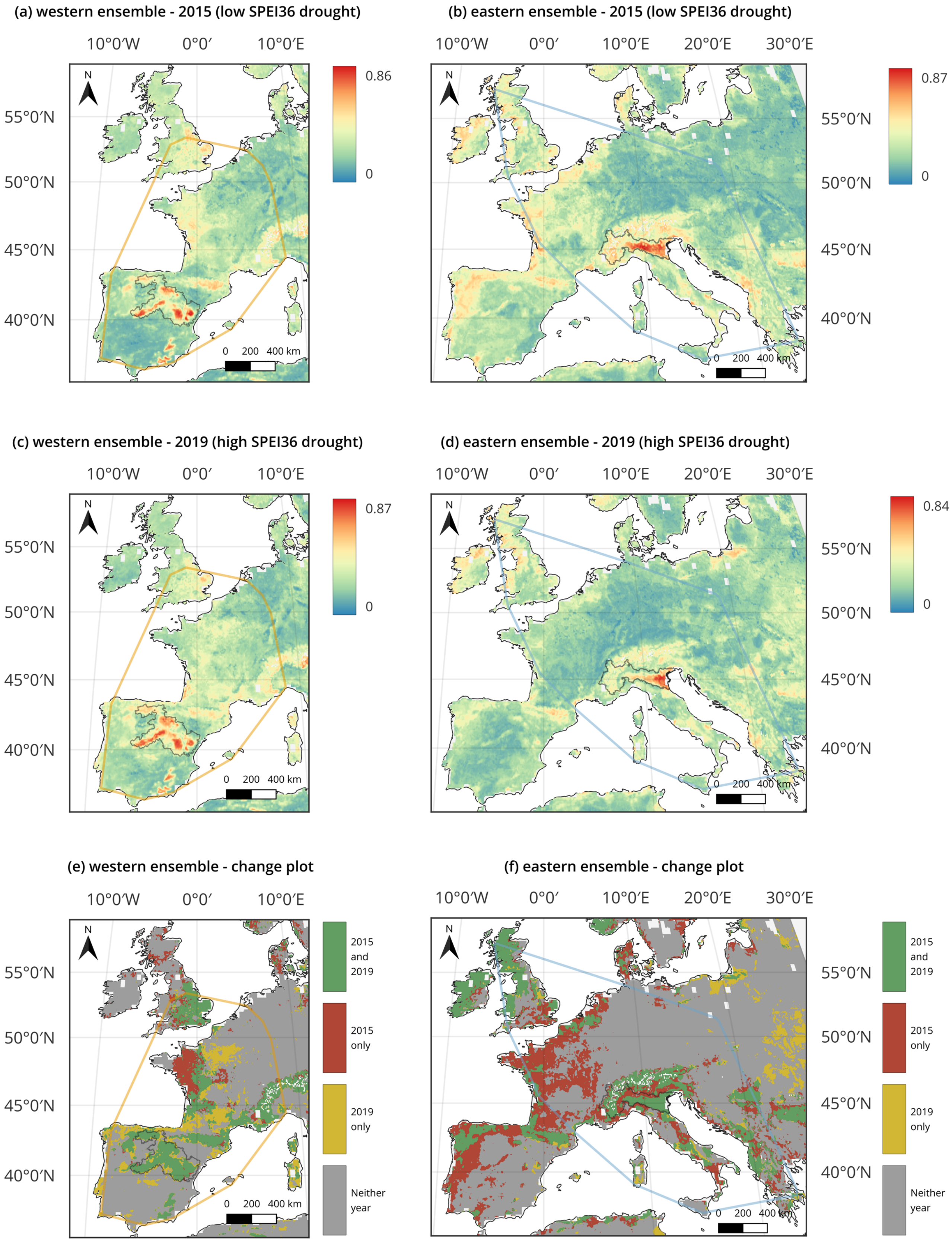


Figure S3.19: ensemble spatial predictions for the western (a, c, e) and eastern (b, d, f) migration routes. Heat maps for years of low (a & b) and high (c & d) SPEI36 drought indicate stopover probability (see keys). Key regions of predicted stopover use are outlined by black borders. For change maps (e & f), colours indicate whether stopovers were predicted across one, both or neither years (see keys). Polygons denote the area in which models were trained. Maps are projected using the Mollweide projection (ESRI: 54009).

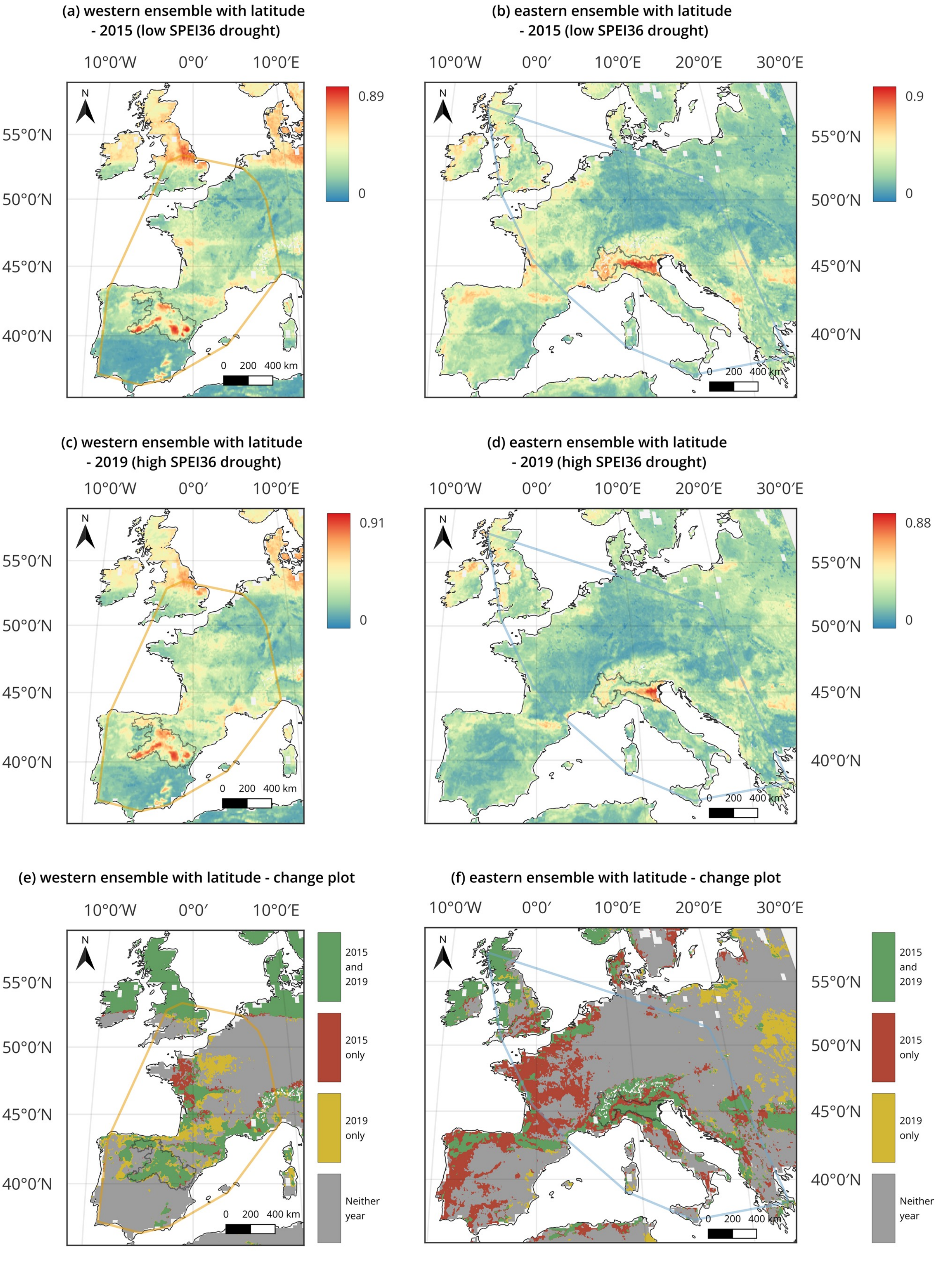


Figure S3.20: ensemble spatial predictions for the western (a, c, e) and eastern (b, d, f) migration routes, with latitude included as a predictor. Heat maps for years of low (a & b) and high (c & d) SPEI36 drought indicate stopover probability (see keys). Key regions of predicted stopover use are outlined by black borders. For change maps (e & f), colours indicate whether stopovers were predicted across one, both or neither years (see keys). Polygons denote the area in which models were trained. Maps are projected using the Mollweide projection (ESRI: 54009).

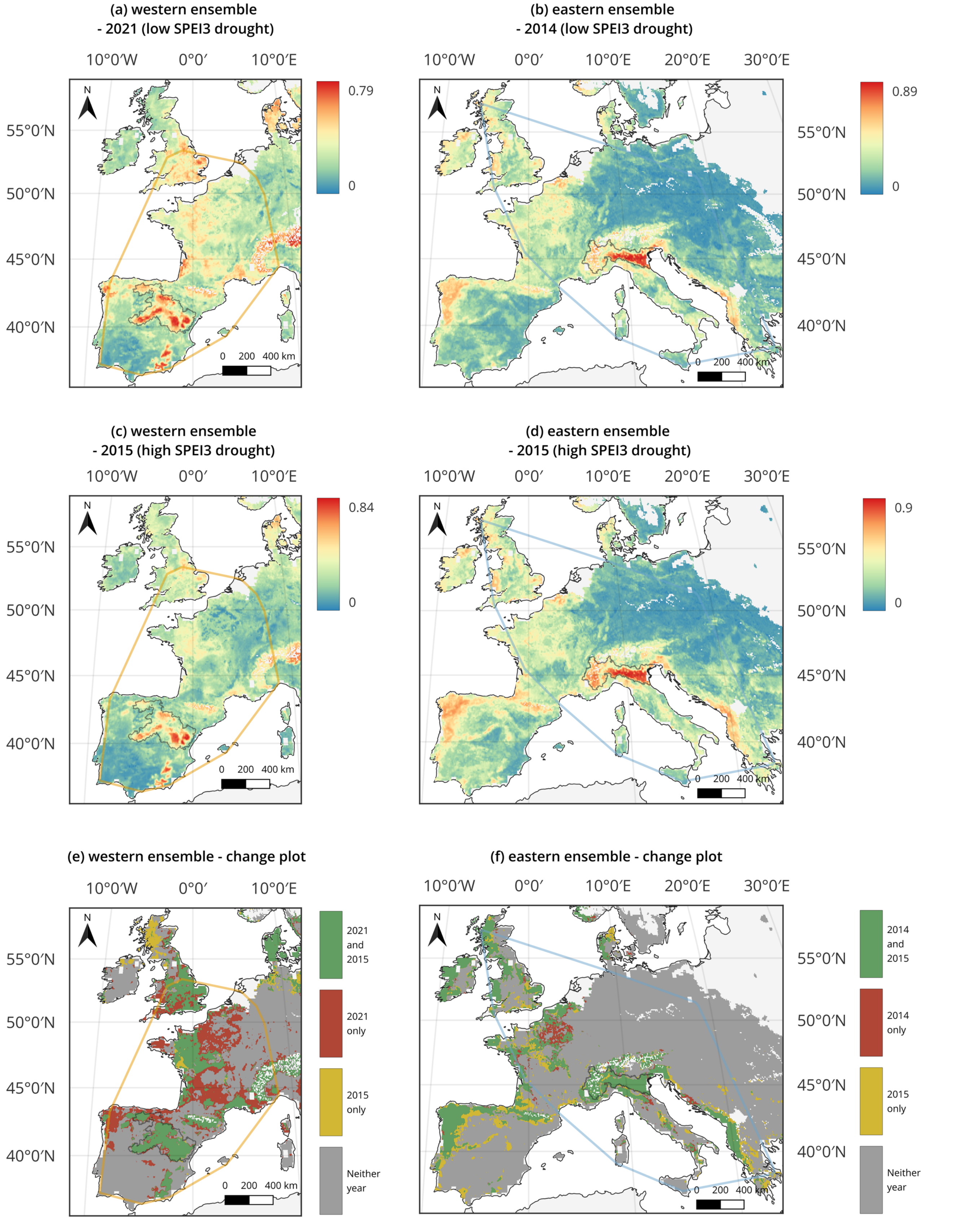


Figure S3.21: ensemble spatial predictions for the western (a, c, e) and eastern (b, d, f) migration routes, based on variables included in sensitivity analyses. Heat maps for years of low (a & b) and high (c & d) SPEI3 drought indicate stopover probability (see keys). Key regions of predicted stopover use are outlined by black borders. For change maps (e & f), colours indicate whether stopovers were predicted across one, both or neither years (see keys). Polygons denote the area in which models were trained. Maps are projected using the Mollweide projection (ESRI: 54009)

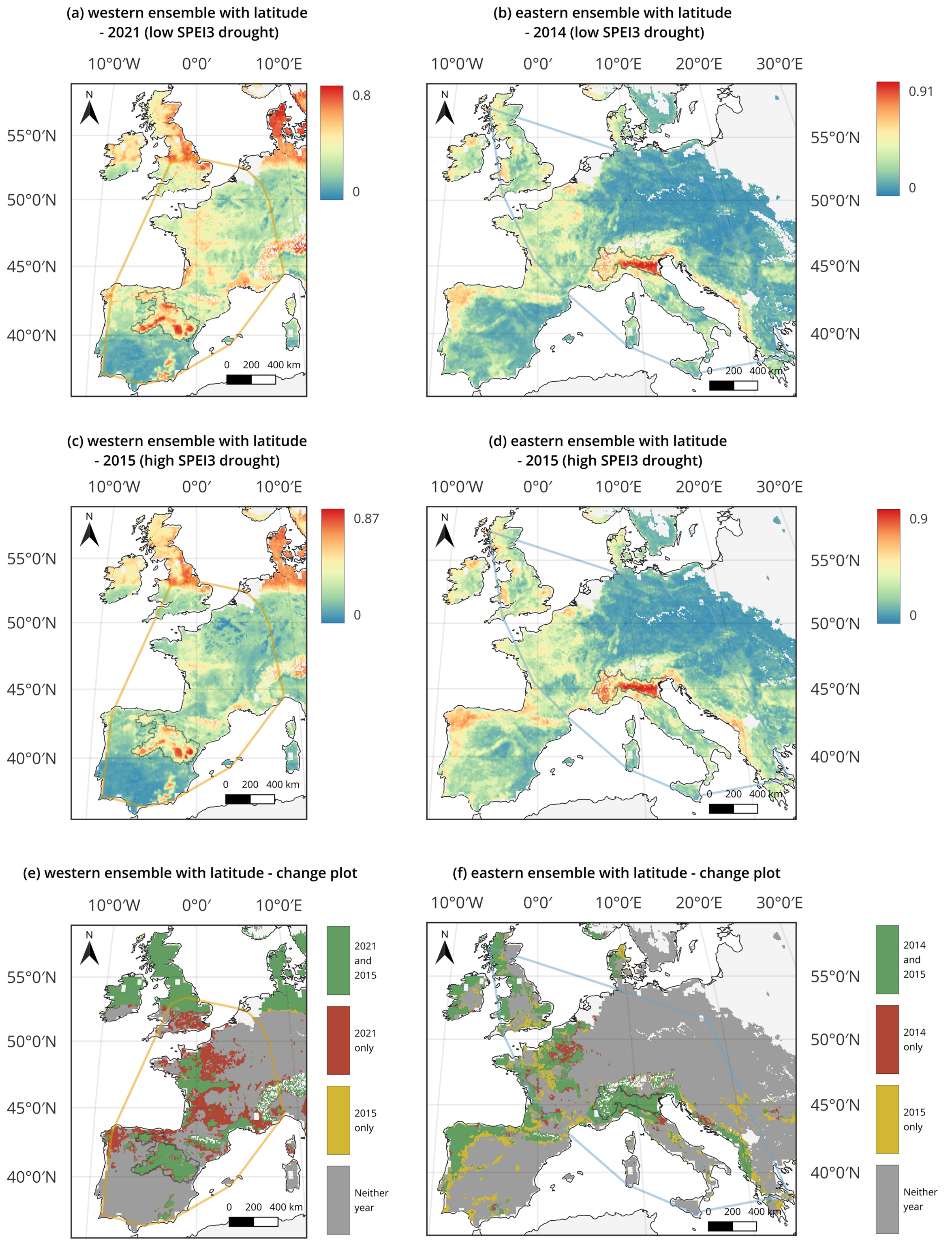


Figure S3.22: ensemble spatial predictions for the western (a, c, e) and eastern (b, d, f) migration routes, based on variables included in sensitivity analyses, plus latitude. Heat maps for years of low (a & b) and high (c & d) SPEI3 drought indicate stopover probability (see keys). Key regions of predicted stopover use are outlined by black borders. For change maps (e & f), colours indicate whether stopovers were predicted across one, both or neither years (see keys). Polygons denote the area in which models were trained. Maps are projected using the Mollweide projection (ESRI: 54009 )
