# Supplementary material for: Drivers of Stopover Distribution Across Two Autumn Migration Routes of a Long‐Distance Afro‐Palearctic Migrant, the Common Cuckoo ( Cuculus canorus )
Source: Ecol Evol. 2026 Aug 4;16(8):e74083. doi: 10.1002/ece3.74083 (PMC13435883; doi:10.1002/ece3.74083)
Supplement: Supplementary file 3 [file ECE3-16-e74083-s003.docx]

## **Appendix S1**

**Atypical stopovers**

No quantitative rule was employed to identify ‘atypical’ stopovers; rather, such occurrences were subjectively removed under the following justifications. The three atypical eastern stopovers within or bordering Iberia (figure S3.1, circles 1 and 2) were made by individuals 161318 (‘PJ’) and 134956 (‘Hennah’), both of whom began on a south westerly trajectory but subsequently switched to south easterly bearings. To prevent these unusual stopovers distorting the area and environmental conditions typical of the eastern route, removal was deemed appropriate. Similarly, the two atypical western stopovers in Austria (figure S3.1, circle 3) were made by individual 134960 (‘Maji’), who began on an eastern trajectory before pursuing a western route. Again, removal was deemed appropriate.

**Collinearity analysis**

Thirteen environmental variables with ecological relevance supported by primary literature were selected *a priori* (table 1). Four additional variables were also considered (table S2.2), but removed following collinearity tests, conducted using the *vicfor* function in the *usdm* package (v.2.1-7; Naimi *et al*., 2014) and *cor* function from the *stats* package (v.4.4.1; R Core Team, 2024). These omissions were total spring precipitation and cumulative winter precipitation (both correlated with aridity), cumulative winter temperature (correlated mean spring temperature), and pesticide use (correlated with cropland), where |*r|* > 0.7 (figure S3.2, tables S2.3 and S2.4). Aridity index was favoured over other correlated variables following Kassara *et al*.’s (2025) recent research highlighting its important effect on stopover use at Mediterranean refuelling sites for several trans-Saharan, insectivorous passerine migrants. Mean spring temperature was favoured owing to its ecological importance (triggering budburst and enhancing larval development in the periods immediately before migration; Macphie *et al*., 2023). Cropland index was preferred to pesticide use for its superior data quality, being derived from high resolution PROBA-V satellite imagery (CLMS, 2019), as opposed to government estimates of nation-wide pesticide use (FAO, 2025).

**Curation of environmental variables**

**Cumulative winter precipitation**. Total winter precipitation for a given year (*W*_y_) in each raster grid cell was calculated using equation 1:

(1)

$$W_{y}=P_{11,y-1}+P_{12,y-1}+P_{1,y}$$

Where *P_11_, P_12_* and *P_1_* represent the total precipitation for November, December and January (respectively), and y is the given year. Cumulative mean winter precipitation (*C*_y_) was then calculated following equation 2:

(2)

$$C_{y}=\sqrt{(\sqrt{W_{y-1}\times W_{y-2}})\times W_{y}}$$

Thus, total winter precipitation of the current year is weighted by the geometric mean winter precipitation of the previous two years. In this way, *C*_y_ puts greatest emphasis on current year’s precipitation, while still reflecting the precipitation of previous years. Successive years of above (or below) average winter rain will therefore be reflected in *C*_y_, even if winter precipitation of the current year (*W*_y_) is typical.

**Cumulative winter temperature**.

Mean winter temperature for a given year (*M*_y_) is calculated following equation 3:

(3)

$$M_{y}=\sqrt{d_{11, y-1} \times d_{12,y-1} \times d_{1, y}}$$

Where *d*_11_, *d*_12_ and *d*_1_ represent mean maximum temperature for November, December and January (respectively), and *y* is the given year. Cumulative mean winter temperature (*T_y_*) is then calculated following equation 4:

$$T_{y}=\sqrt{(\sqrt{M_{y-1}\times M_{y-2}})\times M_{y}}$$

(4)

Thus, cumulative effects of winter temperature over the three years prior are accounted for.

**Mean spring temperature.** Mean spring temperature for each year was calculated by averaging the mean maximum temperature for the months of March, April, May and June.

**Standardised Precipitation-Evapotranspiration Index (SPEI)***.* The SPEI is calculated by summing (1) precipitation and (2) potential evapotranspiration (PET) data over a given period and then calculating the difference (precipitation *minus* PET). Values are then standardised such that the SPEI scale shows the number of standard deviations from normal water balance (values of 0). The normal water balance (values of 0) is determined using data from the entire reference period (1901-present). Thus, the SPEI provides a standardised index of drought which is comparable across space and time. This study includes SPEIs calculated across three time period: 3, 12 and 36 months to capture short-, medium- and long-term drought, respectively. SPEI data was obtained from the Hydro-JULES data depository (Gebrechorkos *et al*., 2023).

**De Martonne aridity index**. The De Martonne aridity index (DMAI) was calculated for each year of the study period, following equation 5:

$$DMAI=P/(T+10)$$

(5)

Where *P* is the mean annual precipitation (mm), and *T* is the mean annual temperature (^o^C).

**Habitat indices**. For the *grassland,* *cropland, shrubland and woodland* indices, fractional land cover layers were first obtained from Copernicus Land Monitoring Scheme (CLMS, 2019). The CLMS depository contains annual land cover data from 2015 to 2019 at 100m resolution. All data was downloaded and averaged for each variable, creating single habitat layers for the whole study period (2011-2021); 100m cells were then aggregated into 10km cells and averaged, using the *aggregate* function of the *stats* package (v.4.4.1; R Core Team, 2024). The creation of single habitat layers for the whole study period was deemed appropriate, given the lack of annual data for some years (2011-14; 2020-21). Whilst finer scale habitat preferences may be concealed by aggregating 100m grid cells, we justify our approach on the grounds that typical cuckoo stopover ranges fall between 5-10km (Williams *et al*., 2016); thus, 10km was deemed an appropriate scale to capture the broader habitat experienced during stopovers. Indeed, higher resolutions (~100m) may equally give an inaccurate representation of the wider habitat used by cuckoos at stopover sites. For example, a cuckoo transmitting from a 100m grid cell of forestry plantation may give the false signal that cuckoos target dense woodland, when the wider 10km square is likely to contain a mix of habitats.

The *wetland* index was derived from the CLMS’s (2018) water and wetness status: a categorical layer denoting the presence (or absence) of water bodies and wetlands at 100m resolution between 2012 and 2018. Permanent water bodies and wetlands were combined with temporary water and wetlands to create a binary raster layer. This layer was then resampled to a resolution of 10km (using the *resample* function in the *terra* package v1.7-78; Hijmans, 2024), with each cells value being the proportion of 100m wetland pixels within the resampled cell.

**Habitat heterogeneity**. To determine the *heterogeneity* of habitat types utilised by cuckoos at an appropriate scale, Shannon diversity values for each 10km cell (*H’*) were calculated using the five habitat indices outlined above:

(6)

$$H^{'}=-\sum_{i=1}^{5} p_{i}\times ln(p_{i})$$

Where *p_i_* is the percentage coverage (index value) of habitat type *i* in each grid cell. Values ranged from 0 to a maximum of 1.6, but were subsequently normalised to a more interpretable scale (0-1) consistent with other habitat indices. Diversity calculations and creation of the resulting *habitat heterogeneity* raster were achieved using the *base* package (v4.4.1, R Core Team, 2024).

**Relative hunting risk**. Buchan *et al*. (2022) quantified and mapped the relative threat of hunting at a 10km resolution, based on the expert opinion of 137 localised respondents across the Palearctic region. This well-informed index of hunting risk is available for small and large birds (relative to the size of a feral Pidgeon) – the former being used here. The data layer was freely obtained from Buchan *et al*. (2022), to which the reader is referred for further details.

**Pesticide use.** Pesticide use was calculated by multiplying total pesticide application per unit area of cropland (kg ha yr ^-2^) by the cropland index raster. Pesticide application data was sourced from the Food and Agricultural Organisation (FAO, 2025) at a national scale for all European countries with available data.

**MaxEnt model tunning and fitting**

5-fold cross-validation tuning was conducted using the *maxnet* function from the *maxnet* package (Phillips, 2021). For the SE route, a linear quadratic hinge (LQH) model with a regularisation multiplier of 1 emerged as one of the joint highest performing models (table S2.5), whilst a linear quadratic product hinge (LQPH) with a regularisation multiplier of 0.5 was highest performing for the SW route (table S2.6). However, concerned that the lack of penalisation on a LQPH model with a regularisation multiplier of 0.5 may result in overfitting (Radosavljevic and Anderson, 2014), we fit models for both routes with feature class = LQH and regularisation multiplier = 1 (the default value; Phillips and Dudík, 2008). This combination remained one of the highest performing models across tunning for both routes (tables S2.5 and S2.6), striking a balance between high performance and model complexity.

**Identifying drought levels across migration routes**

To identify low and high drought years, drought severity was defined using the 3-month SPEI, which captures short-term water deficits during and immediately preceding the migratory period for any given year (see table 1 (main text) for further details). For each route and month, mean SPEI values and the percentage of grid cells with SPEI values < -1 (indicating drought conditions) were calculated using the *stats* (v.4.4.1; R Core Team, 2024) and *dplyr* (Wickham *et al*., 2023) packages; monthly metrics were then averaged to create seasonal drought scores for each year. Drought severity was also calculated for each biogeographical region (as defined by the European Environmental Agency (EEA), 2016) within the migratory study areas, to ensure route-wide trends were not driven by strong droughts in a singular region. For the eastern route, drought coverage was lowest during 2014 and greatest during 2015, while for the western route it was lowest during 2021 and highest during 2015 (figure S3.3); these patterns were largely consistent across all bioregions within each route (figure S3.4). Finally, the above process was repeated using the SPEI36 variable to determine yeas where long-term drought levels were at the highest and lowest. This was done following the result that SPEI36 was a much stronger predictor of eastern stopover distribution than the SPEI3 variable and thus had a greater influence on predicted stopover distribution. For both routes, mean SPEI values and drought coverage was lowest during 2015 and greatest during 2019 (figure S3.5); these patterns were largely consistent across all bioregions within each route (figure S3.6).

**Sensitivity analysis results**

Sensitivity analyses (where total spring precipitation, cumulative winter precipitation, cumulative winter temperature and pesticide use were substituted for aridity, mean spring temperature and cropland) revealed a minor increase in predictive performance (AUC values = + 0.01-0.02, TSS values = + 0.02-0.06) when alternative correlated variables were used (table S2.8), potential resulting from sensitivity models having an additional variable and thus, additional predictive power. Importantly, substituting correlated variables did not substantially alter predictive performance.

Alternative variable selection had only a minimal effect on variable rankings and response curves. For the western sensitivity models, the top five environmental predictors for sensitivity models were the same as primary models, only substituting cumulative winter temperature and cumulative winter precipitation for their correlated pairs (mean spring temperature and DMAI, respectively; figures 2 and S3.9). Sensitivity response curves for substituted variables (figure S3.14) closely followed those of their correlated pairs (figure 3), indicating similar environmental relationships.

For the eastern route, both primary and sensitivity models shared SPEI36, hunting risk and elevation as top predictors (figures 2 and S3.9); however, in contrast to primary models, grassland cover and pesticide ranked among the top five predictors for sensitivity models (figure S3.9), suggesting some sensitivity to variable selection. However, there was minimal variation in permutation importance scores among predictors for the eastern models (figures 2 and S3.14); as such, changes in variable rankings under sensitivity analysis reflect only minor changes (<1%) in permutation importance. Sensitivity response curves for substituted variables (figure S3.14) closely followed those of their correlated pairs (figure 3) except for pesticide use; in contrast to primary response curves indicating limited use of cropland (figure 3), sensitivity curves suggest exposure to greater quantities of pesticides (figure S3.14).

For both routes, spatial predictions from sensitivity models (figures S3.21 and S3.22) were very similar to those of primary models (figures 4 and 5), indicating negligible influence of alternative variable selection on predicted spatial distribution.

**References (including citations in tables S2.2-S2.4)**

Buchan, C., Franco, A.M., Catry, I., Gamero, A., Klvaňová, A. and Gilroy, J.J. (2022) ‘Spatially explicit risk mapping reveals direct anthropogenic impacts on migratory birds’, *Global Ecology and* *Biogeography*, 31(9), pp.1707-1725.

CLMS (2018) *Water and Wetness status 2018 (raster 10 m and 100 m), Europe, 3-yearly*. European Union's Copernicus Land Monitoring Service information. Available at: https://land.copernicus.eu/en/products/high-resolution-layer-water-and-wetness/water-and-wetness-status-2018

CLMS (2019) *Land Cover 2019 (raster 100 m), global, yearly – ver. 3*. European Union's

Copernicus Land Monitoring Service information. Available at: <https://land.copernicus.eu/en/products/global-dynamic-land-cover/copernicus-global-land-service-land-cover-100m-collection-3-epoch-2019-globe#general_info>

Daoud, J.I. (2017) ‘Multicollinearity and regression analysis’, *journal of physics: Conference series*, 949(1), article number: 012009.

Denerley, C., Redpath, S.M., Van der Wal, R., Newson, S.E., Chapman, J.W. and Wilson, J.D. (2019) ‘Breeding ground correlates of the distribution and decline of the Common Cuckoo *Cuculus canorus* at two spatial scales’, *Ibis*, 161(2), pp.346-358.

Dennis, R.L. and Sparks, T.H. (2007) ‘Climate signals are reflected in an 89 year series of British

Lepidoptera records’, *European Journal of Entomology*, 104(4), pp.763-767.

EEA (2016) *Biogeographical regions in Europe*. Available at: [76cb49c587d945a7befc239d65b2376e](https://www.eea.europa.eu/ds_resolveuid/76cb49c587d945a7befc239d65b2376e)

FAO (2025) *Pesticide use dataset* FAOSTAT. Available at: <https://www.fao.org/faostat/en/#data/RP>

Gebrechorkos, S.H., Peng, J., Dyer, E., Miralles, D.G., Vicente-Serrano, S.M., Funk, C., Beck, H.E., Asfaw, D.T., Singer, M.B. and Dadson, S.J. (2023) ‘Global high-resolution drought indices for 1981–2022’, *Earth System Science Data Discussions*, 15(12), pp.5449–5466.

Hijmans, R. (2024) terra: Spatial Data Analysis (v.1.7-78).

Kassara, C., Barboutis, C. and Bounas, A. (2025) ‘Favorable stopover sites and fuel load dynamics of spring bird migrants under a changing climate’, *Climatic Change*, 178(1), article number: 3.

Macphie, K.H., Samplonius, J.M., Pick, J.L., Hadfield, J.D. and Phillimore, A.B. (2023) ‘Modelling thermal sensitivity in the full phenological distribution: A new approach applied to the spring arboreal caterpillar peak.’ *Functional Ecology*, 37(12), pp.3015-3026.

McDermott Long, O., Warren, R., Price, J., Brereton, T.M., Botham, M.S. and Franco, A.M. (2017)

‘Sensitivity of UK butterflies to local climatic extremes: which life stages are most at risk?’, *Journal of Animal Ecology*, 86(1), pp.108-116.

Naimi, B., Hamm, N.A., Groen, T.A., Skidmore, A.K. and Toxopeus, A.G. (2014) ‘Where is

positional uncertainty a problem for species distribution modelling?’*, Ecography*, 37(2), pp.191-203.

Phillips (2021) maxnet: fitting ‘Maxent’ Species Distribution Models with ‘glmnet’ (v.0.1.4).

Phillips, S.J. and Dudík, M. (2008) ‘Modeling of species distributions with Maxent: new extensions and a comprehensive evaluation’, *Ecography*, 31(2), pp.161-175.

Pollard, E. (1988) ‘Temperature, rainfall and butterfly numbers’, *Journal of Applied Ecology*, 25(3),

pp.819-828.

R Core Team (2024) *R: A Language and Environment for Statistical Computing* (v4.1.1).

Radosavljevic, A. and Anderson, R.P. (2014) ‘Making better Maxent models of species distributions: complexity, overfitting and evaluation’, *Journal of biogeography*, 41(4), pp.629-643.

Rigal, S., Dakos,V., Alonso,H., Auniņš, A., Benkő, Z., Brotons, L., Chodkiewicz, T., Chylarecki, P., de Carli, E., del Moral, J.C., Domşa, C., Escandell, V., Fontaine, B., Foppen, R., Gregory, R.,

Harris, S., Herrando, S., Husby, M., Ieronymidou, C., Jiguet, F., Kennedy, J., Klvaňová, A., Kmecl,

P., Kuczyński, L., Kurlavičius, P., Kålås, J.A., Lehikoinen, A., Lindström, Å., Lorrillière, R., Moshøj,

C., Nellis, R., Noble, D., Eskildsen, D.P., Paquet, J., Pélissié, M., Pladevall, C., Portolou, D., Reif,

J., Schmid, H., Seaman, B., Szabo, Z.D., Szép, T., Florenzano, G.T., Teufelbauer, N., Trautmann,

S., van Turnhout, C., Vermouzek, Z., Vikstrøm, T., Voříšek, P., Weiserbs A., and Devictor, V. (2023) ‘Farmland practices are driving bird population decline across Europe’, *Proc. Natl. Acad. Sci. U.S.A.*, 120(21), article number: 2216573120.

Roy, D.B., Rothery, P., Moss, D., Pollard, E. and Thomas, J.A. (2001) ‘Butterfly numbers and

weather: predicting historical trends in abundance and the future effects of climate

change’, *Journal of Animal Ecology*, 70(2), pp.201-217.

Schöll, E.M., Ohm, J., Hoffmann, K.F. and Hille, S.M. (2016) ‘Caterpillar biomass depends on

temperature and precipitation, but does not affect bird reproduction’, *Acta Oecologica*, 74, pp.28-36.

Severns, P.M., Boldt, L. and Villegas, S. (2006) ‘Conserving a wetland butterfly: quantifying early

lifestage survival through seasonal flooding, adult nectar, and habitat preference’, *Journal of Insect Conservation*, 10, pp.361-370.

WallisDeVries, M.F., Baxter, W. and Van Vliet, A.J. (2011) ‘Beyond climate envelopes: effects of

weather on regional population trends in butterflies’, *Oecologia*, 167(2), pp.559-571.

WallisDeVries, M.F. and Van Swaay, C.A. (2006) ‘Global warming and excess nitrogen may induce

butterfly decline by microclimatic cooling’, *Global Change Biology*, 12(9), pp.1620-1626.

Wickham, H., François, R., Henry, L., Müller, K. and Vaughan, D. (2023) *dplyr: A Grammar of Data Manipulation* (v1.1.4).

Williams, H.M., Willemoes, M., Klaassen, R.H., Strandberg, R. and Thorup, K. (2016) ‘Common Cuckoo home ranges are larger in the breeding season than in the non-breeding season and in regions of sparse forest cover’, *Journal of Ornithology*, 157, pp.461-469.
